# Redundant Canonical and Noncanonical *Caenorhabditis elegans* p21-Activated Kinase Signaling Governs Distal Tip Cell Migrations

**DOI:** 10.1534/g3.112.004416

**Published:** 2013-02-01

**Authors:** Eldon C. Peters, Andrea J. Gossett, Bob Goldstein, Channing J. Der, David J. Reiner

**Affiliations:** * Lineberger Comprehensive Cancer Center, University of North Carolina, Chapel Hill, North Carolina 27599; † Department of Biology, University of North Carolina, Chapel Hill, North Carolina 27599; ‡ Department of Pharmacology, University of North Carolina, Chapel Hill, North Carolina 27599

**Keywords:** RhoG, Dock180, ELMO, MIG-2, RAC-2, PAK-2

## Abstract

p21-activated kinases (Paks) are prominent mediators of Rac/Cdc42-dependent and -independent signaling and regulate signal transduction and cytoskeletal-based cell movements. We used the reproducible migrations of the *Caenorhabditis elegans* gonadal distal tip cells to show that two of the three nematode Pak proteins, MAX-2 and PAK-1, function redundantly in regulation of cell migration but are regulated by very different mechanisms. First, we suggest that MAX-2 requires CED-10/Rac function and thus functions canonically. Second, PIX-1 and GIT-1 function in the same role as PAK-1, and PAK-1 interaction with PIX-1 is required for PAK-1 activity; thus, PAK-1 functions noncanonically. The human Pak-Pix-Git complex is central to noncanonical Pak signaling and requires only modest Rac/CDC-42 input. Unlike the human complex, our results suggest that the *C. elegans* Pak-Pix-Git complex requires PAK-1 kinase domain activity. This study delineates signaling network relationships in this cell migration model, thus providing potential further mechanistic insights and an assessment of total Pak contribution to cell migration events.

The p21-activated kinases, or Paks, are a group of Ste20-related kinases that function in signal transduction and regulation of cytoskeletal organization. They are defined by shared kinase domain homology and the ability to be bound and regulated by Cdc42 and Rac small GTPases (Bokoch 2003; Hofmann *et al.* 2004). Rho family small GTPases, including Cdc42 and Rac, are regulated by their guanine nucleotide binding state; guanine nucleotide exchange factors (GEFs) stimulate GTP loading and hence activation, whereas GTPase-activating proteins accelerate the intrinsic GTPase activity to hydrolyze guanosine triphosphate (*i.e.*, GTP) to guanosine diphosphate (*i.e.*, GDP) and inactivate the protein (Wennerberg and Der 2004).

Mammalian Paks are divided into two groups: Group A (Pak1, 2, 3) and Group B (Pak4, 5, 6). Cdc42 or Rac frequently activates Group A Paks. Although they possess a p21-binding domain (PBD) capable of binding Cdc42 and Rac, Group B Paks are not activated upon GTPase binding and probably have functions distinct from the Group A Paks (Arias-Romero and Chernoff 2008). Instead, the Rac or Cdc42 GTPase may be involved in subcellular targeting of the Group B Paks, but Pak activity is promoted by some independent mechanism. Both groups are thought to regulate the cytoskeleton and cell movements, but the Group A Paks are far better studied. In addition to activities mediated by the kinase domain, Group A Paks also can function as a kinase- and Rac-Cdc42-independent scaffold for a complex containing the exchange factor Pix and the G-protein−coupled receptor kinase interactor Git (Loo *et al.* 2004; Parnas *et al.* 2001). Their kinase-independent activities are thought to be GTPase independent, although perhaps the GTPase aids subcellular localization. In addition, kinase-independent but Rac-dependent Pak has been shown to function as a scaffold for PDK and Akt signaling (Higuchi *et al.* 2008).

Group A Paks have been associated with a variety of human diseases, including oncogenesis and metastasis in cancer (reviewed in Kumar *et al.* 2006), Alzheimer’s disease (McPhie *et al.* 2003; Zhao *et al.* 2006), and X-linked nonsyndromic mental retardation (Allen *et al.* 1998; Bienvenu *et al.* 2000; Gedeon *et al.* 2003). This latter function is thought to involve Pak in the context of the Pak-Pix-Git complex rather than canonical Rac-Pak signaling (Kutsche *et al.* 2000). Together, these Pak-based disease etiologies, along with the regulation of cytoskeleton and cell projections by Paks, argue that Paks are critical regulators of cell movements and are important for the understanding of many diseases. However, the extensive redundancy inherent in six mammalian Pak genes suggests that Pak studies in simpler metazoans, which have fewer Paks, could shed important light on total Pak contribution to cell biology.

*Caenorhabditis elegans* contains three Pak proteins (Chen *et al.* 1996; Hofmann *et al.* 2004); PAK-1 shares all known sequence motifs with Group A Paks, PAK-2 is more similar to Group B Paks, and MAX-2, although closest in sequence identity to Group A Paks in the PBD and kinase regions, does not share other N-terminal regulatory sequence motifs typical of Group A Paks. PAK-1 binds CDC-42 and CED-10/Rac and during morphogenesis colocalizes with these GTPases at the plasma membrane of epithelial cells (Chen *et al.* 1996). Loss of MAX-2 causes modest disruptions of axonal pathfinding and loss of PAK-1 greatly enhances these defects, but loss of PAK-1 alone causes no obvious defects (Lucanic *et al.* 2006). Consistent with the frequently observed antagonism between Rac/Cdc42 and Rho, in Drosophila Pak and Rho are antagonistic (Vlachos and Harden 2010), and Pak participates with Rac and Cdc42 in migrations of epithelial sheets (Harden *et al.* 1999). Use of multiple signals and multiple GTPase effectors is complex in growth cone migration (Demarco and Lundquist 2010; Lucanic *et al.* 2006; Lundquist *et al.* 2001; Norris *et al.* 2009; Quinn *et al.* 2008; Shakir *et al.* 2006, 2008; Soto *et al.* 2002; Struckhoff and Lundquist 2003) and epithelial morphogenesis (Gally *et al.* 2009; Patel *et al.* 2008; Zhang *et al.* 2011). Distal tip cell migration, analyzed here, may provide a simpler model for Pak pathway relationships.

In this study we analyze the roles of *C. elegans* Pak proteins in migration of the distal tip cells (DTCs) of the gonad. DTCs are somatic gonadal cells whose migratory path during larval development dictates the shape of the mature gonad (Hedgecock *et al.* 1987; Kimble and Hirsh 1979). Starting ventrally near the anterior/posterior (A/P) mid-point in the body, the two DTCs migrate anteriorly or posteriorly on the surface of the basement membrane, turn dorsally, then turn again to migrate back to the A/P mid-point, thus forming a highly reproducible inverted-U shape. During migration, the DTC remains connected to its gonad arm, and the gonad proceeds with germline development. Because the precise route of DTC migration can be inferred after the fact by the final shape of the mature gonad (Nishiwaki 1999), DTC migration is an excellent system for studying the genetic regulation of cell pathfinding and migration.

Here we describe striking synergy between *max-2* and *pak-1* in DTC migration. Loss of both results in pathfinding errors and incompletely extended gonad arms, suggesting that these redundant Paks are critical for both appropriate directionality of migration and migration itself. Genetically, we show that CED-10/Rac is likely to function upstream of MAX-2, whereas the PIX-1/Pix and GIT-1/Git orthologs are required for PAK-1 branch activity. Similar genetic relationships have been previously described (Lucanic and Cheng 2008), although we arrive at somewhat-different mechanistic conclusions. We also show that the CED-2, -5, /-12 Dock/ELMO noncanonical RacGEF complex is likely to activate CED-10 in DTC migration. Despite the apparent role of PAK-1 in a Pak-Pix-Git complex, which was previously described as being kinase-independent, our data suggest that PAK-1 kinase domain activity is required for PAK-1 branch function. Furthermore, genetic analysis suggests a modest contribution of CED-10/Rac to PAK-1 branch activity, suggesting that unlike previously described, the Pak-Pix-Git complex is partially Rac-dependent. We also identify other events during *C. elegans* development that redundantly require MAX-2 and PAK-1.

## Materials and Methods

### *C. elegans* culturing

Handling, maintenance, and nomenclature of *C. elegans* strains were as described (Brenner 1974; Horvitz *et al.* 1979). All strains were derived from the N2 (Bristol) wild type and were cultured on *Escherichia coli* strain OP50. Animals were grown in a 23° incubator, which we found to be the optimal temperature for bacterially mediated RNAi. A summary of key mutations used in this study is shown in Table 1.

**Table 1:** Mutant alleles used in this study and their effects on protein function

Gene	Allele	Lesion	Proposed Functional Effect	Reference
*pak-1*	*ok448*	Exon 7-10 Δ (out of frame)	Kinase deleted, regulatory intact	Lucanic *et al.* 2006
	*tm403*	Exon 1-7 Δ (in-frame)	Regulatory sequences deleted, kinase domain intact (lf or gf?)	Zhang *et al.* 2011
*max-2*	*nv162*	Exon 1-4 Δ (incl. ATG/starting Met)	Null for full-length isoform	Lucanic *et al.* 2006
	*cy2*	G340E	Loss of kinase function	Lucanic *et al.* 2006
*pak-2*	*ok332*	Exon 1-7 Δ	Putative null	Fujiki *et al.* 2010
*ced-10*	*n1993*	V190G	Reduced function	Reddien and Horvitz 2000
	*n3246*	G60R	Altered function (gf or dn)	Reddien and Horvitz 2000; Shakir *et al.* 2006
*mig-2*	*mu28*	W60 stop	Putative null	Zipkin *et al.* 1997
*pix-1*	*ok982*	Exon 12-14 Δ	Probable strong loss	Lucanic and Cheng 2008
	*gk416*	Exon 1-4 Δ	Probable strong loss	Lucanic and Cheng 2008
*git-1*	*tm1962*	Exon 8-9 Δ	Probable strong loss	Lucanic and Cheng 2008

### Scoring DTC migration phenotypes

Hermaphrodites were grown at 23° and scored at the late L4 stage on 3% agar pads using a Nikon microscope equipped with Differential Interference Contrast (DIC) Nomarski optics. The DTC migration path was inferred from the final position of the two fully developed gonad arms, and for data storage for each animal, the shape of both gonad arms was sketched. For each gonad arm, the entire trajectory was tracked, including Z axis changes. Using this technique we found that we could reproducibly distinguish between folded-over and truncated gonad arms. In previous studies authors suggest that each arm migrates independently of the other, so that each arm constitutes an individual data point (Nishiwaki 1999).

Scoring DTC migration revealed two general phenotypic classes: (1) pathfinding defects, where the DTCs migrated the normal distance but made inappropriate turns; and (2) truncation defects, where the DTC migration stopped immediately after the dorsal turn. In addition, some DTCs exhibited both pathfinding and truncation defects. A DTC was scored as wild type if it extended from the vulva, turned dorsally, then returned to the midline, forming a “U” shape in one plane. Also scored as wild type were gonads with minor shape perturbations that appear at low frequency in all genotypes observed, including the wild type. Included in this category are gonads in whom the distal arm dips ventrally at the end of its migration, distal arms whose migration is slightly short, and minor dorsal extension defects that cause slight ripples in the resulting gonad. We also note that all strains derived from the N2 Bristol wild type had a baseline of 10–15% pathfinding defects, although no truncation defects were observed.

We define “pathfinding” defects as gonads that have made incorrect turns during development. We observed no reproducible patterns associated with specific genotypes, and there were many different ways in which the gonads could mis-migrate, including failures of all turns and most possible inappropriate turns. At low frequency we observed a DTC migrating past the A/P midline, although not a significant distance.

A truncation defect describes gonads with an early termination of migration. This phenomenon can be broken down into three categories: (1) a short arm, where the gonad extends, but makes no dorsal turn; (2) a dorsal “hockey stick,” where the gonad stops just after the dorsal turn; and (3) no arm, where the gonad does not extend at all. The large majority of truncations observed were the “hockey stick” variety, where the DTC makes the dorsal turn but stops. Many truncated gonads were swollen at the distal end, as if there were not sufficient room in the truncated gonad to contain the germline proliferation therein.

The N2 wild-type strain showed minor pathfinding defects at low penetrance. We examined N2 Bristol stocks from several different laboratories and the CB4856 wild isolate from Hawaii and observed defects at average penetrance of 12%. Most defects were minor, but all strains had rare animals with significant pathfinding defects. We analyzed plates made in different laboratories and incubators in different buildings and never saw an appreciable difference in basal defect rates. We hypothesize that this phenomenon is not restricted to the N2 strain and is a general feature of *C. elegans* DTC migration.

### Bacterially mediated RNAi protocol

RNAi methods were derived from described protocols (Kamath *et al.* 2003; Timmons *et al.* 2001). HT115 bacteria, transformed with L4440 plasmid containing coding region for the gene of interest, were plated for no more than 16 hr on LB media containing tetracycline and ampicillin. Overnight cultures in LB plus ampicillin were grown for no more than 12 hr (longer growth could decrease RNAi efficacy). A total of 80 μL of each feeding RNAi culture was spotted onto two NG plates containing 1 mM IPTG and 50 μg/mL carbenecillin. Depending on the fecundity of the strain, four to eight late L4 hermaphrodites were picked to a plate for each feeding RNAi clone. Animals were picked to RNAi plates using mineral oil to minimize transfer of contaminating OP50 bacteria. Animals were transferred after 24 hr to the second plate from the same feeding RNAi culture. L4 hermaphrodite progeny were scored 48 hr after the transfer.

Every experiment included positive and negative controls of *pop-1*- and *gfp*-directed RNAi, respectively. In our hands under optimal conditions, *pop-1*-directed RNAi induced 100% embryonic lethality. Experiments where animals escaped *pop-1*-induced lethality were discarded. All RNAi experiments were compared with *gfp*-directed RNAi baselines to control for possible migration effects of engaging the RNAi response.

### *pak-1 d*ominance test

We tested potential dominance of *pak-1* alleles by comparing *max-2(RNAi)*; *pak-1(tm403)*/*+* with *max-2(RNAi)*; *pak-1(ok448)*/*+* animals. *pak-1* is on the X chromosome, so we performed this experiment by crossing N2 males to *tm403* homozygotes to generate *tm403/0* hemizygous males, which were then crossed into *dpy-8(e130)*; *unc-6(e78*). This second cross was performed on *max-2* RNAi plates, and non-Unc non-Dpy hermaphrodite L4 progeny were scored for DTC migration defects.

### Plasmids used

Bacterially mediated RNAi used genomic or cDNA sequence insertions in the pPD129.36 (a.k.a. L4440) backbone. Those annotated with chromosomal locations were from the Ahringer library (Kamath *et al.* 2001). Pre-existing RNAi plasmids used were: *gfp* (Zand *et al.* 2011), *pop-1* (I-1K04), *pak-1* (X-3E04), *max-2* (II-8F19), *git-1* (X-5I19), *unc-22* (IV-6K06), and *rac-2 (*IV-7L24). Our constructed feeding RNAi plasmids, pEP2.7 (L4440+*ced-10)*: We digested pEL319 (Lundquist *et al.* 2001) with *Bgl*I/*Nco*I to remove *rrf-3*, leaving only *ced-10* cDNA, and then it was Klenow treated and blunt ligated. pEP1 (L4440+*mig-2*): we used DJR611/612 (ATGTCTTCACCGTCGAGGCAG/CGTCGCGCAAATCGAGTTTGG) from *C. elegans* genomic DNA to amplify a 970-bp region between exons 1 and 3 of *mig-2* and cloned it into L4440 digested with *Not*I/*Sal*I.

Markers and experimental plasmids for transgenics, *i.e.*, pMH86 (*dpy-20* (+)), pPD118.33 (P_myo-2_::*gfp*): The pJK590 plasmid (P*_lag-2_*) (Blelloch *et al.* 1999) uses the 3-kb sequence upstream of the *lag-2* start codon (5′ tttttaaattctcat–caaatttgccttt 3′) and contains a mutated *Bsm*I site within the promoter to assist in cloning. pCM15.2 (P*_lag-2_*::*cdc-42*): We amplified *cdc-42* cDNA from plasmid yk1443h8 by using DJR549/550 (ATGCAGACGATCAAGTGCGTCGTCG/TTAGAGAATATTGCACTTCTTCTTC) and cloning it into pCM14.1 (P*_lag-2_*::*chw-1*) digested with *Age*I/*Not*I (V. Muñiz-Medina, C. J. Der, and D. J. Reiner, unpublished data). We mutagenized pCM15.2 with DJR517/518 to create pCM15.3 (P*_lag-2_*::*cdc-42 Q61L*) and mutagenized pCM15.2 with DJR623/624 to generate pCM15.4 (P*_lag-2_*::*cdc-42 S17N*). pCM13.1 (P_lag-2_::*ced-10*): *ced-10* cDNA was amplified from plasmid pEL319 using DJR545/546 (ATGCAAGCGATCAAATGTGTCGTCG/ TTAGAGCACCGTACACTTGCTCTTTTTGG) was cloned into pCM14.1 digested with *Age* I/*Not* I. We mutagenized pCM13.1 with DJR513/514 to create pEP3.2 (P*_lag-2_*::*ced-10 Q61L*). pEP12.1 (P*_lag-2_*::*pak-1(+)*): we amplified *pak-1* cDNA from yk1619a12 by using DJR631/629 (AGGCTTGCCAAAATGAAAGCTTTCTCATCG/TTATGAGTTGCTAGCTTCGGCGATGC) and cloning it into pCM14.1 digested with *Kpn*I/*Not*I. We mutagenized pEP12.1 with DJR678/679 to generate the disrupted Pix binding mutant pEP12.2 (P*_lag-2_*::*pak-1(234A,235A)*) and mutagenized pEP12.1 with DJR674/675 to generate the kinase dead mutant pEP12.3 (P*_lag-2_*::*pak-1(324R)*). pEP15.1 (*P_let-858_*::*pak-1*(+)): We digested pPD118.25 with *Nhe*I to remove *gfp* and then religated to create pEP10.1. We amplified *pak-1* cDNA from yk1619a12 by using DJR631/629 (as described previously) and cloned into pEP10.1 digested with *Kpn*I/*Spe*I. Sequencing primers for pPD118.25 were DJR655/656 and for pJK590mut were DJR654/660. The sequences of plasmids and oligonucleotides are available upon request.

### Transgene generation

Markers (concentrations) used included: pA2132 (*unc-119* (+)) at 50 ng/μL; pMH86 (*dpy-20* (+)) at 20 ng/μL; pPD118.33 (P*_myo-2_*::*gfp*) at 5 ng/μL; P*_lag-2_*::*ced-10* (*or cdc-42*) at 5 ng/μL; and P*_lag-2_*::*pak-1 at* 5 or 10 ng/μL.

### Mutations used

We used the following mutations: *pak-1*(*ok448*), *pak-1*(*tm403*), *max-2*(*nv162*), *max-2*(*cy2*), *mig-2*(*mu28*), *pix-1*(*ok982*), *pix-1*(*gk416*), *git-1*(*tm1962*), *ced-10*(*n1993*), *ced-10*(*n3246*), *ced-1*(*e1756*), *ced-5*(*n1812*), *ced-12*(*n3261*), *unc-33*(*e204*), *hIn1*[*unc-54*(*h1040*)], *unc-29*(*e193*), *dpy-24*(*s71*), *unc-43*(*n498n1179*), *dpy-8*(130), *unc-6*(*e78*), *unc-119*(*ed3*), *dpy-20*(*e1282*). Before performing double loss-of-function analysis, we outcrossed *pak-1* alleles *ok448* and *tm403* for 10× and 5×, respectively). *max-2* alleles *nv162* and *cy2* were both outcrossed 4×.

### Primers for genotyping

To detect deletions for outcrossing the alleles we used the following primers: *pak-1(ok448*): DJR684.f1 CAGTACACAAAACCGAAAGAGGAGG, DJR685.f2 GTTGGTAAAAGTTAGGGATGACCCC, DJR686.r1 TTCTCTTACCTTGAACAACAAAGTCATGG; *pak-1(tm403)*: DJR636.f1 ATGAGGGCATGTAATACACAAGTACCG, DJR637.f2 TTGCATGCTTATTCTCACGCATCACC, DJR638.r1 GAATCTCTTCCAGGGAAGTCGGG; *max-2(nv162)* (Lucanic *et al.* 2006): nv162.f1 CCGGCAGGAAGACTATATGACTC, nv162.r1 CACAAAGAGGGAAGAAGATCCTC, nv162.r2 CCTTCTTCTGATCGGCAAGACTG; *max-2(cy2)* (Lucanic *et al.* 2006): *cy2*.f1 CGGCAGTGTTGTCTCCACAACAT, *cy2*.r1, GCCGAGCAGCACGTTGTCACTCT, *cy2* eliminates a *BspE* I site in the amplicon; git*-1(tm1962)*: DJR667.f1 CGATCTCAGTTCTCCCGAAAGG, DJR668.f2 GCAAAATGAGGAGCTGACAAAGTG, DJR669.r1 GGTGGCATAACTCTTTCTGGAGT; p*ix-1(gk416)*: DJR661.f1 CCCCGCAAAAGAGACCCAGAG, DJR662.f2 TCGGAAAAGGAGTACATGCAAAGC, DJR663.r1 GAAACAGTGAGCTACTCGCCCC; and *pix-1(ok982)*: DJR664.f1 GGACTCAGTGAGCCACTCCGG, DJR665.f2 CATCCCCCAGGAAACTCGGAT, DJR666.r1 CAATGCGGGGCTCGAACC.

## Results

### PAK-1 and MAX-2 redundantly control DTC migration

**Figure 1:**
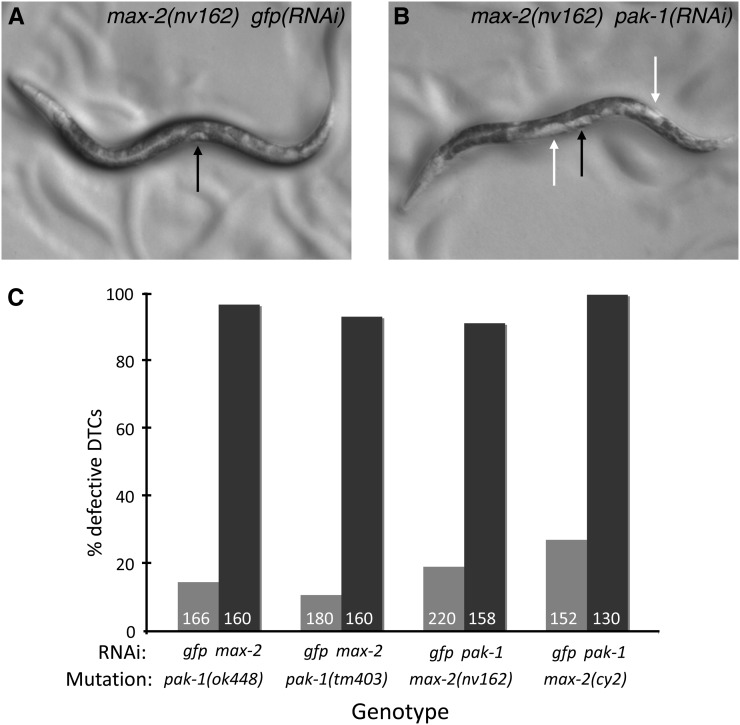
*max-2* and *pak-1* are redundant for DTC migration. Bright-field photomicrographs of (A) *max-2(nv162)* and (B) *max-2(nv162)*; *pak-1(RNAi)* late L4 animals. In both animals anterior is left and ventral is down. A black arrow indicates the semicircular clear patch formed by the developing vulva and uterus at the late L4 stage, and white arrows indicate inappropriate clear patches caused by gonad overlap. (C) All pair-wise combinations of *max-2* and *pak-1* disruption show strong synthetic DTC migration and gonad shape defects. Number in columns is the number of gonad arms scored. *pak-1* single mutants on negative control *gfp*-directed RNAi were not significantly different from the wild type (5–15% defective in all experiments, see Figure 3B for example), whereas *max-2* loss of function mutations may cause DTC migration defects that are slightly elevated; cy2 but not *nv162* on *gfp(RNAi)* was significantly different than both *ok448* and *tm403* (*P* < 0.05). For all *gfp vs. pak-1* or *max-2* RNAi comparisons (paired columns), *P* < 0.0001 by Fisher’s exact test. No error bars are shown because these are pooled assays.

To analyze the functions of Pak kinases during development, we disrupted Pak activity by RNAi-targeting one Pak gene in a background where another Pak gene was mutated. At a morphological level, double loss-of-function animals appeared wild type except for one phenotype; greater than 90% of animals displayed light patches in various parts of the body between the pharynx and anus (Figure 1, A and B). This patchy phenotype is often seen with defects in migration of the gonadal DTCs (Nishiwaki 1999). Normally, the two DTCs of the somatic gonad migrate in mirror image U-shapes. Each arm of the mature gonad occupies a lateral half of the body cavity, whereas the intestine, which appears dark in the dissecting microscope, occupies the other half. Thus, because animals on the plate generally are viewed from the side, in the wild type one observes the uninterrupted dark band of the intestine running the length of the animal, and the clear gonad arms are not visible. However, in the event of defective DTC pathfinding, portions of the gonad can overlap, occupying both the left and right planes, from the lateral perspective, and thus displace the intestine. This intestinal displacement results in clear patches that are easily detected by dissecting microscope, and this phenotype has been shown to be strong enough to isolate mutants in genetic screens (Nishiwaki 1999).

By DIC/Nomarksi microscopy, we recorded highly penetrant DTC migration defects in *max-2*; *pak-1* double loss-of-function animals compared with few defects in the single mutant (Figure 1C). We observed a wide range of pathfinding defects, but with one exception (see *Pak controls both DTC pathfinding and migration execution*, below) no class of defect was more representative than another, and both the anterior and posterior gonad arms were affected equally. We analyzed two alleles of each gene in double loss-of-function combinations by targeting the other Pak gene with RNAi; all observed mutation-RNAi combinations conferred strong synthetic DTC migration defects, with no quantitative or qualitative differences between the genetic combinations (Figure 1C). As negative controls, single-mutant animals subjected to *gfp*-directed RNAi were not significantly different from animals grown without RNAi.

Alleles used in this study are listed in Table 1. *pak-1(ok448)* deletes most of the kinase domain and disrupts subsequent reading frame and should therefore be null for PAK-1 kinase function. *pak-1(tm403)* deletes most of the N-terminal regulatory sequences, including the PBD, the autoinhibitory domain, the Pix-binding site, the acidic region and many of the potentially regulatory PXXP SH2-binding sites, but frame is retained and a functional kinase should be made. Such a truncated protein could result in a gain-of-function or toxic product (see *pak-1(tm403) may be weakly semidominant*, below). *max-2(nv162)* deletes the first four exons of the gene, including the start codon, and thus represents a null for full-length pak function. *max-2(cy2)* is a point mutation altering a conserved residue in the kinase domain, suggesting that it causes strong kinase loss of function (Lucanic *et al.* 2006). We note that both *pak-1* and *max-2* express multiple isoforms due to alternative splicing or use of alternative promoters, including shorter isoforms altogether lacking the N-terminal regulatory regions (wormbase.org WS228). Furthermore, mammalian Group A Paks have been shown to have kinase-independent functions (see *Putative PAK-1 partners GIT-1 and PIX-1 are redundant with MAX-2*, below). Thus, mutations that eliminate certain domains of the protein may still retain developmental functions, and none of the mutations characterized thus far is likely to be null for all gene functions.

### *pak-1(tm403)* may be weakly semidominant

The *pak-1(tm403)* allele deletes the N-term regulatory sequences in-frame and, due to de-regulation, might produce a gain-of-function or otherwise toxic protein. Thus *tm403* might be predicted to be semidominant. We tested this prediction genetically. *gfp(RNAi)*; *pak-1(tm403)*/*+* and *gfp(RNAi)*; *pak-1(ok448)*/*+* hermaphrodites had similar, wild-type levels of DTC migration defects. However, *max-2(RNAi)*; *pak-1(tm403)*/*+* and *max-2(RNAi)*; *pak-1(ok448)*/*+* were significantly different, with almost 27% of *max-2(RNAi)*; *pak-1(tm403)/+* gonad arms having DTC migration defects compared with 17% in the control (Table 2; *P* < 0.05, Fisher’s exact test). These observations suggest that *pak-1(tm403)* might encode a toxic, semidominant protein. As additional support for this hypothesis, we were unable to transgenically rescue *max-2(RNAi)*; *pak-1(tm403)* DTC migration defects with wild-type PAK-1 protein, whereas we could rescue *max-2(RNAi)*; *pak-1(ok448)* (see *PAK-1 is cell autonomous and requires kinase activity and PIX-1-interaction sequences*, below). Despite this predicted difference in consequences of the two *pak-1* alleles, *pak-1(tm403)* and *pak-1(ok448)* homozygotes on *max-2*-directed RNAi conferred DTC migration defects of similar strength (Figure 1C). Interestingly, the *ced-10(n1993)* hypomorphic allele and *n3246* weak gain-of-function allele (Shakir *et al.* 2006) on *pak-1(RNAi)* also have roughly similar affects on DTC migration [Figure 3; *ced-10* null is maternal effect lethal (Kinchen *et al.* 2005) and was not used for this analysis for technical reasons]. Because it is outside of the scope of this study, we have not pursued further the potentially toxic nature of *pak-1(tm403)*.

**Table 2:** *pak-1(tm403)* but not *pak-1(ok448)* is weakly dominant

Genotype	% DTC Defect (N)
*gfp(RNAi)*; *pak-1(ok448)*/+	10.2 (168)
*gfp(RNAi)*; *pak-1(tm403)*/+	12.5 (160)
*max-2(RNAi)*; *pak-1(ok448)*/+	17.3 (228)
*max-2(RNAi)*; *pak-1(tm403)*/+	26.5 (148)

*ok448*/*+* or *tm403*/*+* animals were tested for dominant effects by plating on *gfp*-directed (control) or *max-2*-directed (experimental) RNAi and assessing frequency of DTC migration defects. *tm403/+*; *max-2(RNAi)* is significantly different than *tm403/+*; *gfp(RNAi)* (*P* < 0.05, Fisher’s exact test). DTC, distal tip cells; N, number of gonad arms scored.

### Paks control both DTC pathfinding and migration execution

**Figure 2:**
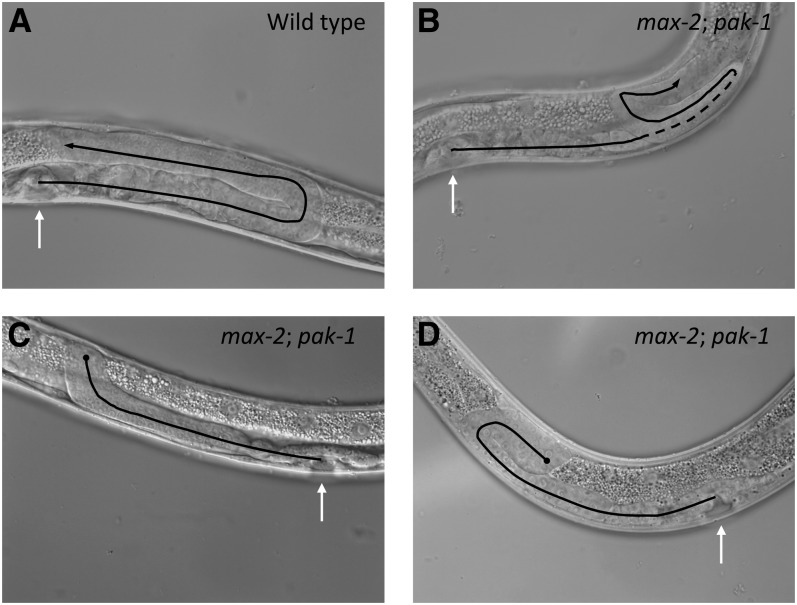
Loss of *max-2* and *pak-1* causes errors in both pathfinding and migration completion. Shown are DIC photomicrographs of single gonad arms, lateral view. In all panels, late L4 animals were used and white arrows point to the developing vulva. Black lines track the path of DTC migration inferred by terminal gonad shape, and the dotted line indicates track of the gonad that is out of the focal plane. Black lines end with an arrow for a completed migrations and a circle for a truncated migrations. (A) Wild-type, U-shaped gonad arm. (B−D) *max-2(nv162)*; *pak-1(ok448)* animals from double-heterozygote parents. (B) Gonad arm with a typical pathfinding error, where the distal arm reversed. (C) Truncated gonad arm with a typical, “hockey stick” morphology, where the distal turn is made but subsequent migration fails. (D) An atypical truncated gonad arm where some migration back toward the vulval is accomplished. For both (C) and (D), typical widening of the gonad at the distal end is visible.

We observed two general classes of gonad morphology defects in *max-2*; *pak-1* double loss-of-function animals: pathfinding and migration execution (defects that are referred to here as “truncation.”) We hypothesize that these defects reflect two distinct functional roles for *max-2* and *pak-1* in DTC migration. First, a broad variety of pathfinding defects were observed (a typical example is shown in Figure 2B, but all possible permutations were observed at low frequency). No single defect was representative of the *max-2*; *pak-1* double, but instead many possible misguidance defects were represented, and aberrant DTC turns were observed at every stage of DTC migration. We hypothesize that these phenotypes reflect a general requirement for redundant *max-2* and *pak-1* function in communicating positional information from the extracellular environment into the migrating cell.

Second, a large subset of mutant animals had prematurely terminated DTC migrations, where migration was arrested/truncated just after the DTC made the dorsal turn halfway through the wild-type migration path (Figure 2, C and D). Generally, the resulting gonads were of otherwise normal shape, although we observed bulging of the gonad arm toward the distal end of the gonad (Figure 2D). Table 3 displays the frequencies of the two classes of phenotypes. We note that the high frequency of DTC truncations likely obscures underlying pathfinding defects, thereby depressing our estimate of pathfinding defects in genotypes with frequent truncation defects. We speculate that without the truncation defect, most DTCs would still have pathfinding defects. The fertility of truncated gonad arms could not be assessed, because (1) they were frequently paired in the same animal with non-truncated gonad arms, and (2) we observed severe sterility in all animals in which both Pak pathways were disrupted (see *PAK-1 and MAX-2 are required for fertility*, below), even in animals in which neither gonad arm was truncated.

**Table 3:** Disruption of both *pak-1* and *max-2* branches is required for significant DTC truncation defects

Row	Genotypes	% Pathfinding	% Truncation	N
1	N2 on GFP	11.8	0.0	160
2	*pak-1(ok448)*; *gfp(RNAi)*	13.3	0.0	158
3	*pak-1(ok448)*; *max-2(RNAi)*	29.4	73.1	160
4	*pak-1(tm403)*; *gfp(RNAi)*	11.0	0.0	154
5	*pak-1(tm403)*; *max-2(RNAi)*	1.0	91.8	146
6	*max-2(nv162)*; *gfp(RNAi)*	14.6	1.2	158
7	*max-2(nv162)*; *pak-1(RNAi)*	25.0	80.7	140
8	*max-2(cy2)*; *gfp(RNAi)*	26.1	1.5	130
9	*max-2(cy2)*; *pak-1(RNAi)*	5.0	97.1	140
10	*ced-10(n1993)*; *gfp(RNAi)*	38.1	18.1	160
11	*ced-10(n1993)*; *pak-1(RNAi)*	14.3	92.2	154
12	*ced-10(n3246)*; *gfp(RNAi)*	38.7	42.5	106
13	*ced-10(n3246)*; *pak-1(RNAi)*	3.0	98.0	100
14	*pix-1(ok982)*; *gfp(RNAi)*	16.3	0.0	80
15	*pix-1(ok982)*; *max-2(RNAi)*	1.3	96.3	80
16	*git-1(tm1962)*; *gfp(RNAi)*	7.5	0.0	80
17	*git-1(tm1962)*; *max-2(RNAi)*	12.5	81.3	80

Each gonad arm evaluated was independently scored for defects in pathfinding or truncation, so in principle the pathfinding and truncation columns can total greater than 100% (*e.g.*, see row 9). Greater frequency of truncation defects correlates with lower frequency of pathfinding defects, presumably because defects in distal arm migration were more frequent than defective proximal arm migration, and thus truncated DTC migrations obscure underlying pathfinding defects. There is some overlap between categories (*i.e.*, truncated gonad arms can be mis-migrated) and thus total percent defect can be greater than 100% (*e.g.*, rows 7, 9, 11, 13). DTC, distal tip cells; N, number of gonad arms scored.

Although a small minority of truncated gonad arms failed to make the dorsal turn, generally the point of migration arrest was reproducible; most truncated DTCs truncated immediately after the dorsal turn, resulting in a shape like a hockey stick. To test whether DTC migration rate was diminished, we scored DTCs at the young adult stage rather than the usual L4 stage to give DTCs additional time to migrate, but we observed no differences in the frequency of truncated gonads, nor were truncated gonads migrated any farther. Therefore, we speculate that loss of *max-2* and *pak-1* results not in generally compromised cell migration, which we predict would result in gonads truncated at many points but rather disrupts response to a specific migratory cue, *i.e.*, that which guides the DTC back to the A/P midpoint of the animal near the developing vulva.

Below we describe genetic analyses of other components of the MAX-2 and PAK-1 signaling pathways. In all cases in which we disrupted the two redundant pathways, we observed accompanying pathfinding defects and truncated migrations (Table 3). This observation suggests that the same genetic pathways control both processes.

Previous work described altered DTC morphology in *pak-1* single mutants (Lucanic and Cheng 2008). We scored *pak-1(ok448)* and *pak-1(tm403)* mutants blindly and were unable to detect reproducible DTC morphology defects relative to the wild type. We do not know the source of this discrepancy.

### CED-10/Rac and the Dock/ELMO RacGEF complex function upstream of MAX-2

**Figure 3:**
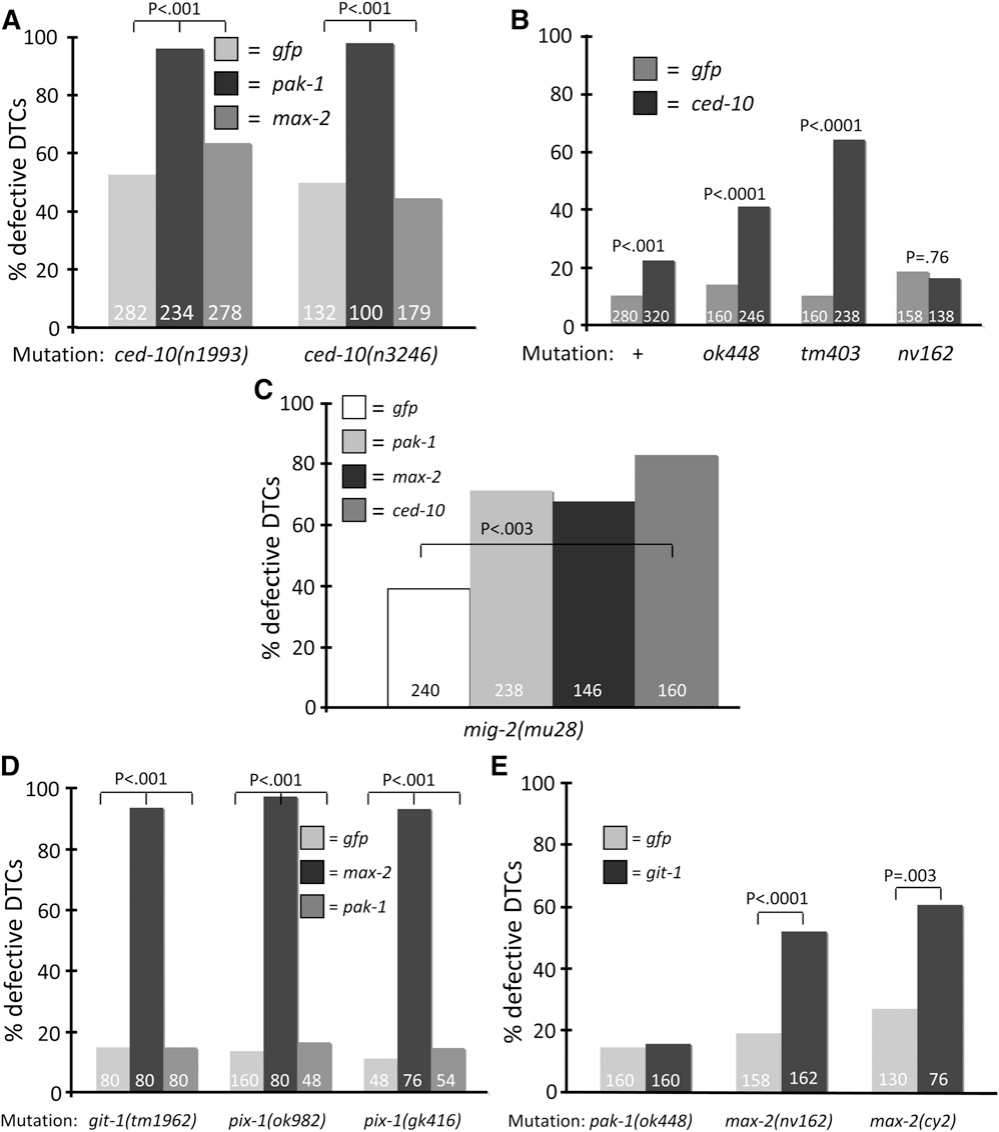
CED-10 acts primarily through MAX-2 and not PAK-1. Number of gonad arms assayed is shown in each column. (A) Both *ced-10* alleles examined had significant baseline DTC defects and showed strong synthetic defects with *pak-1*- but not *max-2*-directed RNAi. (B) The wild-type, *pak-1* and *max-2* animals were grown on *ced-10*-directed RNAi. RNAi of *ced-10* induces an additional defect in the N2 wild-type background that is significant (*P* < 0.001), but in all of our experiments the effects of *ced-10*-directed RNAi were significantly weaker than defects seen for *ced-10* hypomorphic *n1993* and weak gain-of-function *n3246* alleles. The two *pak-1* alleles but not *max-2(nv162)* are synthetic with *ced-10(RNAi)*. (C) RNAi of *pak-1*, *max-2*, or *ced-10* enhance the previously described DTC migration defect of *mig-2(mu28)* (*P* < 0.003), arguing that *mig-2* functions in parallel to the two Pak pathways. (D) Mutations in *git-1* and *pix-1* are strongly synthetic with RNAi targeting *max-2* but not *pak-1*. (E) Mutations in *max-2* but not *pak-1* are synthetic with RNAi targeting *git-1*. Statistics were generated via Fisher’s exact test. No error bars are shown because these are pooled assays.

Based on extensive mammalian work, we hypothesized that CED-10/Rac would be required to activate PAK-1 and/or MAX-2, and we tested this model genetically by pair-wise knockdown of putative pathway components. Both *ced-10* mutations examined had synthetic interactions with *pak-1* but not *max-2*-directed RNAi (90% *vs.* 45–60%; Figure 3A). *pak-1(ok448)* and *pak-1(tm403)* animals had moderate synthetic defects when grown on *ced-10*-directed RNAi, whereas *max-2(nv162)* was not enhanced (~40% and 60% *vs.* <20%; Figure 3B). *ced-10*-directed RNAi was only modestly effective in all contexts, even though we included *rrf-3* sequences in the RNAi vector to hypersensitize animals to *ced-10*-directed RNAi (Simmer *et al.* 2002).

An atypical Rac-specific Dock/ELMO GEF complex has been described in both mammals and nematodes, and the corresponding CED-2/5/12 complex activates CED-10/Rac in cell corpse engulfment. Single mutants for each of these genes, as well as *ced-10*, have been described as having moderately penetrant DTC migration defects (Gumienny *et al.* 2001; Reddien and Horvitz 2000; Wu and Horvitz 1998). We analyzed mutations in the Dock/ELMO complex in combination with loss of *pak-1* or *max-2*. Double-mutant combinations between *ced-5* or *ced-12* and *pak-1* yielded animals with the patchy bright-field phenotype indicative of DTC migration defects, whereas double-mutant combinations between *ced-5* or *ced-12* and *max-2* were no different than single mutants (Table 4). We validated these observations with *pak-1* and *max-2*-directed RNAi: loss of *ced*-2, *ced-5*, or *ced-12* conferred strong synthetic DTC migration defects in combination with *pak-1(RNAi)* but not *max-2*(*RNAi)*. These results suggest that the RacGEF complex required for CED-10 activation in cell corpse engulfment also functions in the same pathway as CED-10 in DTC migration because it has the same genetic interactions with *max-2* and *pak-1*. Given published data about the Dock/ELMO complex, we propose that this RacGEF activates CED-10 to control DTC migration, and our genetic data argue that CED-10 then signals principally through MAX-2/Pak. That CED-10/Rac signals through MAX-2 but not PAK-1 was previously deduced, although the analysis was not extended to the Dock/ELMO complex and did not include multiple alleles of *pak-1*, *max-2*, and *ced-10* (Lucanic and Cheng 2008).

**Table 4:** The Dock/Elmo RacGEF complex is redundant with *pak-1* but not *max-2*

Genotypes	+	*ced-10(n1993)*	*ced-5(n1812)*	*ced-12(n3261)*	*ced-1(e1735)*
+	+	Fertile, wk patchy	Fertile, wk patchy	Fertile, wk patchy	Fertile
*pak-1(ok448)*	Fertile	Sterile, str patchy	Sterile, str patchy	Sterile, str patchy	Fertile
*pak-1(tm403)*	Fertile	Sterile, str patchy	Sterile, str patchy	Sterile, str patchy	Fertile
*max-2(nv162)*	Fertile	Fertile, Unc	Fertile, wk patchy	Fertile, wk patchy	Fertile
*max-2(cy2)*	Fertile	Fertile, Unc	ND	ND	ND

Like *ced-10(n1993)*, *ced-5(n1812)* and *ced-12(n3261)*, which encode components of the Dock/Elmo RacGEF complex, caused synthetic DTC migration and fertility defects in combination with *pak-1*, but not *max-2*. Animals were scored by inspection under dissecting microscope: “Str” is “strong,” “wk” is “weak,” and “Unc” is “uncoordinated.” *ced-1(e1735)*, which regulates cell corpse engulfment through a different pathway, was included as a negative control. As described, *max-2* mutations also cause a severe uncoordinated phenotype in the *ced-10(n1993)* background (Lucanic and Cheng 2008), but not in *ced-5* and *ced-12* backgrounds (our observations), arguing that a GEF other than CED-2/5/12 functions to activate CED-10 in neurite outgrowth. ND, not determined.

The synergy between loss of CED-5/12 or CED-10 and loss of PAK-1, but not MAX-2, suggests that the CED-10/Rac protein signals primarily through MAX-2, whereas PAK-1 functions in a parallel signaling branch. However, one observation complicates this simple interpretation. CED-10/Rac was previously described as being required for DTC migration (Wu and Horvitz 1998), and we found that *ced-10(n1993)* or *ced-10(n3246)* grown on *gfp*-directed RNAi had DTC migration defects well above background levels (Figure 3A). Moderate defects in DTC migration were previously described for mutations disrupting the Dock/ELMO RacGEF (Gumienny *et al.* 2001; Reddien and Horvitz 2000; Wu and Horvitz 1998). We similarly observed mismigrated DTCs in *ced-2(n1994)*, *ced-2(e1752)*, *ced-5(n1812)*, *ced-12(tp2)*, and *ced-12(n3261)* backgrounds. Loss of these proteins had similar effects on DTC truncation (Table 3) (Gumienny *et al.* 2001; Reddien and Horvitz 2000; Wu and Horvitz 1998). The contrast in frequency of gonad arm truncation in *pak-1* or *max-2* single mutants compared with *ced-2*, *-5*, *-10*, and *-12* mutant animals leads us to hypothesize that CED-10 and its GEF are required in both Pak pathway branches, though probably to differing degrees.

### CED-10 signals though MAX-2, but the contribution of other GTPases is unclear

The robust synthetic phenotype of *max-2*; *pak-1* double loss-of-function animals provides an opportunity to dissect the regulation of these Pak proteins *in vivo* and thereby understand their function in cell migration. Paks are generally thought of as effectors of Rho family small GTPases, specifically Rac and Cdc42. *C. elegans* contains a single Cdc42 homolog, CDC-42, and two Rac homologs, CED-10 and RAC-2. CDC-42 *and* CED-10 are very highly conserved with their mammalian counterparts and are absolutely conserved in the Switch 1 effector-binding region, but RAC-2 contains divergent residues in the Switch 1 region. Based on genetic analysis, CED-10 appears to be the major Rac homolog in *C. elegans*, whereas RAC-2 may play a minor, redundant role (Lundquist *et al.* 2001). In addition, *mig-2* encodes a putative homolog of mammalian RhoG, a Cdc-42- and Rac-related but functionally distinct member of the Rho family that generally interacts with effectors different than those of Rac or Cdc42 (deBakker *et al.* 2004; Wennerberg and Der 2004).

*rac-2(RNAi)* caused weak synergy in the *max-2(nv162)* but not *max-2(cy2)* backgrounds (24.6% and 13.8%, respectively, *P* < 0.02). The *rac-2(ok326)* mutation did not confer single mutant DTC defects or synthetic interactions in combination with *pak-1* or *max-2* RNAi (combination with *pak-1* or *max-2* RNAi, 9.4 and 12.2%, respectively). Previous studies (Lucanic and Cheng 2008; Lundquist *et al.* 2001) suggest that RAC-2 plays a modest role in DTC migration, but our results are weak or negative. This discrepancy in a relatively mild phenotype could be due to experimental differences.

*cdc-42*-directed RNAi confers strong embryonic lethality, and surviving animals are grossly defective in gonad organization as well as showing disruption of early somatic gonad polarity (Welchman *et al.* 2007). Previously, *cdc-42*-directed DTC tissue-specific RNAi did not perturb DTC migration on its own. However, *cdc-42+pix-1* but not *cdc-42+max-2* double DTC-specific RNAi caused a weak synthetic phenotype (Lucanic and Cheng 2008). The strength of this effect was weaker than the strong synthetic phenotype observed between *pak-1* and *max-2*, perhaps due to variability in RNAi efficacy from gene to gene. Alternatively, perhaps contribution of CDC-42 to MAX-2 is modest. But the conclusion was that CDC-42 plays a role in parallel to the Pak-Pix-Git complex, perhaps upstream of MAX-2 (Lucanic and Cheng 2008).

To test this hypothesis with a different methodology, we expressed mutant CDC-42(T17N) protein specifically in DTCs using the *lag-2* promoter, which drives expression in DTCs and not surrounding cells (Blelloch *et al.* 1999). This 17N mutation is well characterized in Ras and Rho small GTPases as conferring potent dominant-negative activity, and the main caveat with this reagent is that it could be overly strong by sequestering GEFs required for activation of other Rho family members in addition to CDC-42. DTC-specific CDC-42(T17N) caused no DTC migration defects and was not pursued further (data not shown).

We also tested the MIG-2/RhoG contribution to Pak-mediated DTC migration. RhoG does not bind and activate Pak proteins in mammalian cells (Wennerberg *et al.* 2002), but by yeast two-hybrid assay MIG-2/RhoG may interact with MAX-2/Pak (Fujiki *et al.* 2010). Along with the previously observed moderate DTC migration defect in *mig-2* null mutant animals (Lundquist *et al.* 2001), we found that loss of *max-2*, *pak-1*, or *ced-10* weakly enhanced *mig-2* mutant defects (Figure 3C), but not with the very strong synergy observed between the *max-2* and *pak-1* pathway branches. Although in general *ced-10(RNAi)* efficacy was modest, *pak-1*- and *max-2*-directed RNAi conferred strong loss-of-function phenotypes. Thus, the weak interaction between *mig-2* and *pak-1* or *max-2* argues that *mig-2* probably does not play a significant role in either of these pathways, but rather is involved in a different aspect of DTC migration. This observation is noteworthy because MIG-2/RhoG has been described as functioning upstream of the Dock/ELMO RacGEF that activates CED-10/Rac in nemotodes and mammalian apoptotic cell corpse engulfment (deBakker *et al.* 2004) and thus might have been predicted to functioning similarly to this group of proteins. To summarize, MIG-2/RhoG functions in DTC migration, but does not have clearly interpretable genetic interactions with other signaling components.

**Figure 4:**
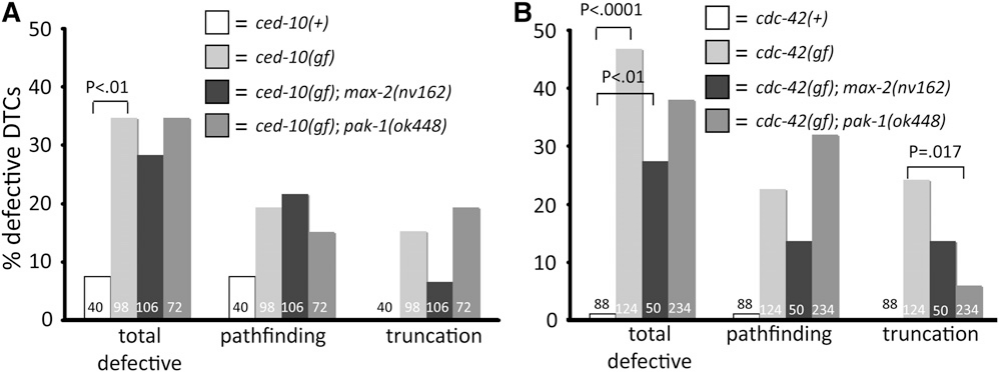
Activated CED-10/Rac and CDC-42 disrupt DTC migration. (A) DTC-directed expression of gain-of-function (gf), mutationally activated CED-10(Q61L) causes pathfinding and truncation defects. (B) DTC-directed expression of gain-of-function, mutationally activated CED-10(Q61L) also causes pathfinding and truncation defects. Loss of either *pak-1* or *max-2* fails to rescue the activated CED-10 defects, whereas the loss of *pak-1* suppresses the truncation but not pathfinding defect of activated CDC-42, and loss of *max-2* modestly suppresses both defects of activated CDC-42. Statistics were generated via Fisher’s exact test, and only significant differences are shown. No error bars are shown because these are pooled assays.

If molecules function in a linear pathway, ectopic activation of the upstream component might allow genetic dissection of pathway colinearity. We expressed wild-type or Q61L-mutated CED-10 specifically in DTCs using the *lag-2* promoter. Q61L mutations or their equivalent activate all Rho and Ras small GTPases by inactivating their intrinsic GTPase catalytic activity, thus locking the protein in the GTP-bound active state. Ectopic CED-10(Q61L) caused defective DTC migrations (Figure 4A), arguing that mutationally activated CED-10 protein has biological activity in migrating DTCs. However, loss of *pak-1* or *max-2* failed to abrogate the activated CED-10 phenotype (Figure 4A), arguing that CED-10 does not exclusively use a single Pak effector pathway, but rather contributes to both, consistent with our arguments based on the aforementioned *ced-2/-5/-10/-12* single mutants.

Similarly, ectopic expression of CDC-42(61L) caused DTC migration defects. Alternatively, CED-10 or CDC-42 could engage effectors other than Paks when ectopically expressed in DTCs, but endogenous protein does not do so. This result may seem to contradict our observation that CDC-42(dn) causes no DTC defect, but we argue that ectopic CDC-42 is capable of engaging Pak effectors and may do so elsewhere during development. PAK-1 loss weakly suppressed the total DTC defect but not specifically pathfinding or truncation, while MAX-2 loss weakly suppressed the truncation defect (Figure 4B). We hypothesize that ectopic, activated CDC-42 can engage both Pak proteins simultaneously, and thus can only be suppressed weakly by loss of single Paks.

### Putative PAK-1 partners GIT-1 and PIX-1 are redundant with MAX-2

Our pair-wise genetic analysis suggests that PAK-1 has a modest requirement for CED-10/Rac activation. This conclusion is consistent with a previously described, partially GTPase-independent function of Group A Paks in cell culture. In addition to its role as a Rac/Cdc42 effector, Pak can function as a scaffold for a protein complex that includes Git1 and Pix (Loo *et al.* 2004). This Pak-Pix-Git complex may not require GTPase input for activation, though Cdc42 and Rac are thought to help target the complex to discrete subcellular compartments, and thus contribute somewhat to optimal complex activity. It is also notable that the mammalian Pak-Pix-Git complex does not require activity of the Pak kinase domain. The signaling output of this complex is unclear, though clearly biologically important (Zhang *et al.* 2011).

The *C. elegans* genes *git-1* and *pix-1* are well-conserved orthologs of Git1 and Pix, respectively (Dyer *et al.* 2010; Lucanic and Cheng 2008). To test whether the putative GTPase-independent role of PAK-1 in DTC migration is GIT-1- and PIX-1-dependent, we used pair-wise genetic knockdowns like those used to characterize PAK-1 and MAX-2 parallelism. We found that outcrossed strains containing *git-1(tm1962)*, *pix-1(gk416)*, or *pix-1(ok982)* conferred no DTC defects alone (or on *gfp*-directed RNAi; Table 3) and were strongly synthetic with *max-2(RNAi)* but not *pak-1(RNAi)*. Conversely, mutants for *max-2* showed strong synthetic defects in combination with *git-1*-directed RNAi, but *pak-1* mutants did not (Figure 3, D and E). Furthermore, mutants for *ced-10* were synthetic with *git-1(RNAi)* (data not shown). These results indicate that *git-1* and *pix-1* are redundant with *max-2* and *ced-10* but not *pak-1*, and therefore we hypothesize that PAK-1 functions as part of a Pak-Git-Pix complex described in other systems.

### PAK-1 is cell autonomous and requires kinase activity and PIX-1-interaction sequences

**Figure 5:**
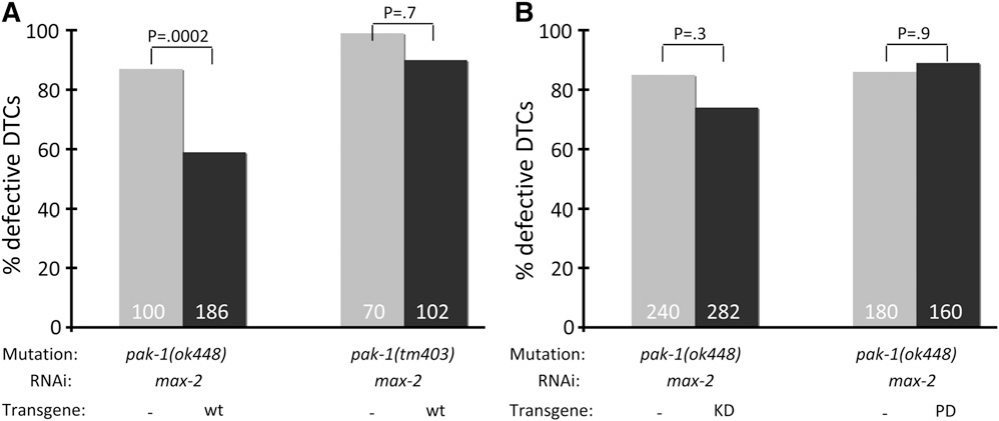
PAK-1(+) but not PAK-1(KD) or PAK-1(Pix dead) rescue PAK-1 null. (A) DTC-directed expression of *pak-1(+)* (isoform A) cDNA partially rescues the DTC migration defect of *pak-1(ok448)* but not *pak-1(tm403)* animals grown on *max-2*-directed RNAi. (B) Neither the KD nor pix-interaction dead *pak-1* cDNAs were able to rescue the DTC migration defect of *pak-1(ok448)* animals grown on *max-2*-directed RNAi. For rescue experiments, array-bearing animals were scored in parallel to nonarray-bearing siblings as an internal control. Statistics were generated via Fisher’s exact test. No error bars are shown because these are pooled assays.

Because a major function of mammalian Pak proteins is to regulate the cytoskeleton, we hypothesized that PAK-1 functions cell autonomously in the DTCs to control their pathfinding and extension. To test this model, we expressed the wild-type *pak-1*A isoform cDNA behind the *lag-2* promoter, which drives expression in DTCs but not surrounding cells (Blelloch *et al.* 1999). We crossed P*_lag-2_*::*pak-1(+)*-containing transgenes marked with P*_myo-2_*::*gfp* into the *pak-1(ok448)* background, which we predict to be null for PAK-1 kinase function. We then subjected the strain to *max-2*-directed RNAi and scored array-bearing *vs.* nonarray-bearing animals (the latter provided an internal control for RNAi efficacy and a baseline for statistical comparison to sibling array-bearing animals). The DTC-directed expression of the *pak-1(+)* cDNA, injected at 5 ng/mL, rescued the synthetic DTC migration defect with *max-2*, although not fully (Figure 5A). To test whether transgene dose was relevant, we also injected at 10 ng/mL, but the rescue results were similar to those at the lower concentration. Therefore, we conclude that a significant portion of PAK-1 function in DTC migration is cell-autonomous, as predicted. We speculate that rescue is incomplete because PAK-1 functions in a multiprotein complex, where stoichiometry may be important for function. Thus, perhaps transgenic overexpression of PAK-1 titrates crucial PAK-1 binding proteins away from the complex, thus compromising function and preventing full rescue. Alternatively, perhaps not all PAK-1 function is cell autonomous, or a different isoform provides some critical *pak-1* function in the DTCs.

We similarly tested P*_lag-2_*::*pak-1(+)* rescue of *pak-1(tm403)*, the in-frame truncation of the PAK-1 N-terminal regulatory sequences that is weakly dominant (see above). We found that DTC-directed expression of *pak-1(+)* failed to rescue *pak-1(tm403)* (Figure 5A). The transgene used was the same used for rescuing *pak-1(ok448)*, so we are confident that wild-type protein is expressed. This result reinforces our hypothesis that *pak-1(tm403)* encodes a toxic, perhaps dominant-negative or gain-of-function protein product.

We further used DTC-directed rescue of PAK-1 to test the molecular requirements of PAK-1 in the putative Pak-Git-Pix complex. If PAK-1 functions as part of such a complex, the Pix-binding domain of PAK-1, which is highly conserved with its mammalian counterparts, should be required for rescue. Therefore, we constructed a P*_lag-2_*::*pak-1(R234A*, *P235A)* “Pix dead” plasmid, guided by previous binding studies of human Pak1 and Pix interactions, in which residues of PAK-1 essential for Pix-binding were mutated (Daniels *et al.* 1999). Using the same assay as for *pak-1(+)* rescue, we found that the “Pix dead” construct failed to rescue the synthetic *max*-2*(RNAi)*; *pak-1(ok448)* DTC migration phenotype (Figure 5B), arguing that PAK-1 binding to PIX-1 is necessary for PAK-1 function in DTCs. Together with the requirement for *pix-1* and *git-1* for *pak-1* branch activity, this result indicates that PAK-1 functions in a complex with PIX-1 and GIT-1 to regulate DTC migration.

Previous mammalian studies suggest that the kinase domain of Pak is dispensable for Pak function with the Pak-Git-Pix complex and the Pak scaffolding activity for phosphatidylinositol 3 kinase and PDK (Higuchi *et al.* 2008). Therefore, we predicted that DTC-directed expression of *pak-1* cDNA with a mutated kinase domain would still be able to rescue the *max-2(RNAi)*; *pak-1(ok448)* synthetic DTC migration defect. We constructed a P*_lag-2_*::*pak-1(K324R)* “kinase dead” (KD) plasmid, guided by previous biochemical and functional assays of human Group A Pak proteins (Manser *et al.* 1997; Sells *et al.* 1997). Surprisingly, we found that DTC-directed expression of *pak-1(K324R)* failed to rescue (Figure 5B). Therefore, we hypothesize that the Pak-Pix-Git complex requires Pak kinase activity to perform its function in DTC migration.

### Pak proteins are required for embryonic morphogenesis

To further characterize Pak-dependent DTC migration defects, we built pair-wise double mutant strains with mutations in *pak-1* (*ok448* and *tm403*) and *max-2* (*nv162* and *cy2*). All allelic combinations were lethal or subviable and therefore impossible to maintain in culture, with severely compromised fertility (see *PAK-1 and MAX-2 are required for fertility*, below) and frequent embryonic morphogenesis defects. This latter defect was not examined in great detail, but cursory inspection revealed embryos in which the epithelium had failed to enclose, thus resulting in gross disorganization of tissue types and gut cells on the exterior of the embryo (Soto *et al.* 2002). We cannot rule out defects in cell fate, but differentiated pharynx and gut were visible. Rare surviving embryos that hatched had variable surface bulges consistent with morphogenetic defects in ventral enclosure or elongation (Chisholm and Hardin 2005). In addition, these rare, surviving double-mutant animals had severe locomotion defects, whereas *pak-1* single-mutant locomotion was wild type, and *max-2* single-mutant locomotion was only mildly defective (Lucanic *et al.* 2006). The lethality and locomotion defects were maternally rescued; double-homozygous mutant F_2_ progeny from a double-heterozygote parent could be readily selected based on the patchy phenotype from DTC migration defects. These patchy animals moved well and had no morphogenetic defects, although they had few or no progeny. We also observed maternal rescue of morphogenetic and locomotion defects when the parent was homozygous mutant for one Pak, and heterozygous mutant for the other Pak, arguing that both *max-2* and *pak-1* are maternally rescued. We conclude that early developmental events like embryonic morphogenesis and axon pathfinding rely on maternal PAK-1 and MAX-2 contribution, whereas later developmental events like DTC migration rely on zygotic gene product.

During these experiments, we found that only one of the four possible pair-wise double mutants yielded viable progeny, albeit at a very low rate. *max-2(cy2)*; *pak-1(tm403)* animals had 2−3 progeny per generation, enough that the strain could be maintained but not frozen. Double mutant animals for other allelic combinations were isolated, but the strains were inviable. However, upon picking many double-mutant animals for each genotypic combination, rare healthier clones of animals arose after a variable number of generations, and the resulting strains were qualitatively more robust. These healthier clones presumably resulted from spontaneous second site mutations that compensated for the severity of the *max-2*; *pak-1* double-mutant embryonic lethality and sterility. Alternatively, perhaps background enhancer mutations were lost, though outcrossed strains were used for the constructions. Unlike the previous Pak mutant study (Lucanic and Cheng 2008) we did not analyze double-mutant DTC phenotypes of these healthier isolates because we were concerned about the potential caveats of additional mutations in unknown genes. Because the healthier clones arose at relatively high frequency, we predict that a large group of genes can be mutated to weakly ameliorate the *max-2*; *pak-1* embryonic lethality and sterility.

The fact that *cy2*; *tm403* can be cultured without second site mutations whereas other allelic combinations could not argues that these two alleles are not null (see above). We note that *pak-1(tm403)* and *max-2(cy2)* have the most severe degree of gonad arm truncation when in combination with RNAi of the redundant Pak gene (Table 3, lines 5 and 9, respectively). Perhaps this observation indicates that both *tm403* and *cy2* encode proteins that are toxic for full-length DTC migration but are less severe than putative null alleles for embryonic morphogenesis. Alternatively, unlike *max-2(nv162)*, which disrupts the translational start of the predicted long but not short isoform, *max-2(cy2)* disrupts kinas domain function, presumably in both isoforms. So perhaps the MAX-2 kinase domain is required in multiple isoforms during DTC migration, but not as strongly required for embryonic development and fertility.

**Figure 6:**
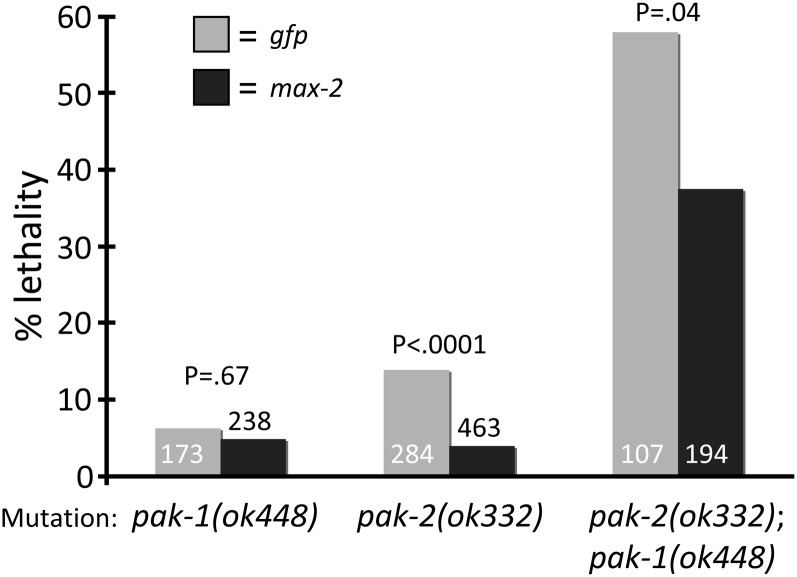
*C.elegans* Pak proteins (PAK-1, MAX-2, and PAK-2) contribute to embryonic development. *pak-1* and *pak-2* function redundantly in embryonic development, and *max-2(RNAi)* suppresses the *pak-2* mutant defect alone or in conjunction with *pak-1*. Lethality in wild-type backgrounds is not shown here but is generally <0.5%. Each pair of columns includes *gfp* or *max-2*-directed RNAi. Sample size indicated in columns. Statistics were generated via Fisher’s exact test. No error bars are shown because these are pooled assays.

PAK-1 is a clear ortholog of mammalian Group A Paks (Pak1, 2, 3), with all known mammalian sequence motifs conserved in the *C. elegans* protein (Chen *et al.* 1996; Hofmann *et al.* 2004). By sequence analysis, MAX-2 is Group A-like, but stereotypical Group A Pak sequence motifs are missing, notably the PXXP SH2 domain recognition motifs and the PIX binding domain (Lucanic *et al.* 2006). Additionally, the MAX-2 PBD, required for binding by activated Rac or Cdc42, is less conserved than that of PAK-1 and the mammalian Group A Pak proteins. *C. elegans* also contains a protein, *pak-2*, that is an ortholog of mammalian Group B Pak proteins (Hofmann *et al.* 2004; Lucanic *et al.* 2006). To examine the total contribution of Pak proteins to embryogenesis, we analyzed single, double, and triple Pak loss of function. We observed that *pak-2(ok332)* single mutants and *pak-1(ok448)* single mutants had significant but relatively low-level embryonic lethality (*P* < 0.0001; Figure 6). We constructed a *pak-2(ok332)*; *pak-1(ok448)* double-mutant strain, and observed significantly more lethality than either the *pak-1* or *pak-2* single-mutant strains (*P* < 0.0001). Growth of *pak-1(ok448)* single-mutant animals on *max-2*-directed RNAi caused no change in baseline lethality, but growth of *pak-2(ok332)* single mutant or *pak-2(ok332)*; *pak-1(ok448)* double-mutant animals on *max-2*-directed RNAi significantly suppressed lethality compared with the *gfp*-directed RNAi control group (*P* < 0.0001 and *P* = 0.04, respectively; Figure 6). Together, these observations suggest that *pak-1* and *pak-2* are redundant for embryonic morphogenesis and fertility, and perhaps that *max-2* antagonizes *pak-2* in this process. Since *pak-2* did not contribute to DTC migration, we did not further pursue *pak-2* analysis.

Additionally, *pak-2(ok332)*; *pak-1(ok448)* double-mutant animals were substantially smaller than wild type as confirmed by blind phenotypic scoring. Neither *pak-1(ok448)* nor *pak-2(ok332)* single mutant animals were of abnormally small stature, suggesting that *pak-1* and *pak-2* are redundant for regulation of body stature. Body stature is controlled by TGF-β signaling (Savage-Dunn 2005), so perhaps PAK-1 and PAK-2 function in that process.

### PAK-1 and MAX-2 are required for fertility

We observed that *max-2*; *pak-1* double-mutant animals were mostly sterile. We similarly observed that *max-2* or *pak-1* single-mutant animals grown on RNAi targeting the reciprocal Pak-encoding gene were mostly sterile. Aside from aberrant pathfinding and shortened gonad arms, these gonads looked mostly normal by DIC. The only defect we observed was that many of the mature oocytes were smaller than their wild-type counterparts. Late L4 single mutant animals picked to RNAi, whose progeny would be sterile, were themselves quite fertile and remained so throughout their adult life, suggesting that the point in germline development in which both *pak-1* and *max-2* are required is before the late L4 stage, or the Pak proteins perdure.

To determine the cause of the extensive sterility, we fixed whole animals at various stages from L4 to mid-adult and stained them with DAPI to visualize DNA. We thus analyzed all pair-wise *max-2*; *pak-1* mutant-RNAi combinations and observed no genotypic differences. As controls, we compared double loss of function to single mutants grown on bacteria expressing *gfp*-directed dsRNA. General gonad architecture in the double mutants was less well organized than in *gfp*-directed RNAi controls, particularly in truncated gonad arms (we could occasionally compare truncated and nontruncated in the same animal). However, in most gonad arms we observed the typical wild-type pattern of distal mitotic proliferation, followed by a clear transition zone, followed by the meiotic region of the gonad and oocyte arrest in diakinesis (Greenstein 2005; Kimble and Crittenden 2005).

**Figure 7:**
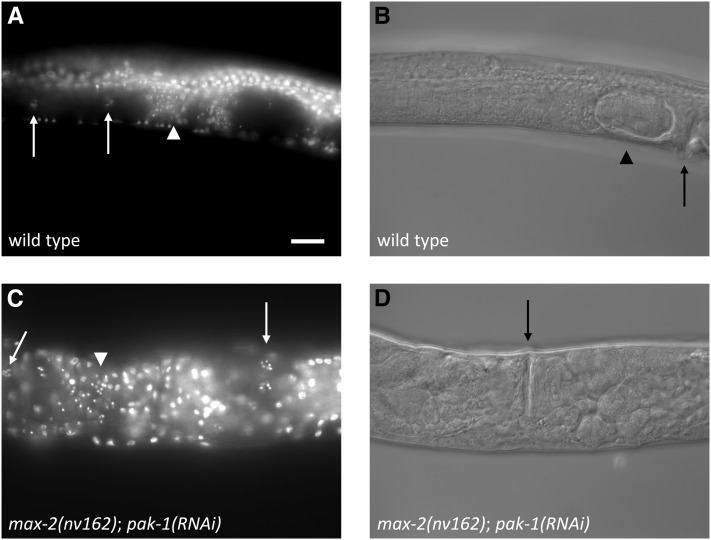
Loss of *max-2* and *pak-1* disrupts spermatogenesis and perhaps other aspects of fertility. (A, B) Wild-type animal. (C, D) *max-2(nv162)*; *pak-1(RNAi)* animal. Both animals are shown from a nearly ventral view. DAPI fluorescence (A, C) and DIC (B, D). The white arrowheads indicate the location of sperm nuclei, white arrows indicate the cluster of bivalents in mature oocytes, black arrows indicate the vulva, and the black arrowhead indicates a fertilized embryo in its eggshell. The double Pak loss of function animal (below) shows sperm nuclei (scattered, small, intense spots) in the left-hand spermatheca, whereas the right-hand spermatheca is empty and no sperm nuclei are visible. Scale bar is 20 μm.

We observed that many gonad arms in mature adults completely lacked sperm nuclei in the spermathecae. This phenotype was incompletely penetrant; although the majority of gonad arms lacked sperm, animals in which one arm had sperm whereas the other did not were relatively common, and we observed several animals in which both gonad arms had sperm in the spermathecae (Figure 7). Given that sufficient animals had plentiful sperm, this putative spermatogenesis defect alone is not enough to explain the sterility observed in double mutant animals. If it were, we would expect isolated animals to have large, “jackpot” broods, a phenomenon we never observed. To determine whether we could rescue the sterility with wild-type sperm, we crossed into double loss-of-function animals with wild-type males, but failed to increase the tiny number of progeny.

We observed that among gonad arms with plentiful sperm and mature oocytes, only rarely did we find a fertilized embryo in the uterus. Not surprisingly, such embryos were observed only in gonad arms with sperm. In contrast, the uteri of control animals at a comparable stage were usually replete with fertilized, developing embryos.

We have thus identified a spermatogenesis defect caused by loss of *max-2* and *pak-1*, but we do not believe that this defect is the sole source of sterility in these animals. Instead, we speculate that loss of *max-2* and *pak-1* could compromise the motility of sperm. *C. elegans* sperm are amoeboid, and active crawling behavior is conducive to fertilization. Consistent with this possibility, we also observed that the sperm in double loss-of-function animals were frequently more diffusely localized than in single mutants, with sperm frequently found in the uterus in spite of the absence of fertilized eggs there (Figure 7). But therefore a sperm motility defect is not sufficient to explain the degree of sterility, suggesting additional defects. We conclude that the sterility phenotype of *max-2*; *pak-1* animals is likely to be complex, comprising a partial spermatogenesis defect, perhaps a sperm migration defect, and presumably additional defects. Consequently, only rarely is an animal able to produce a handful of fertile progeny.

## Discussion

**Figure 8:**
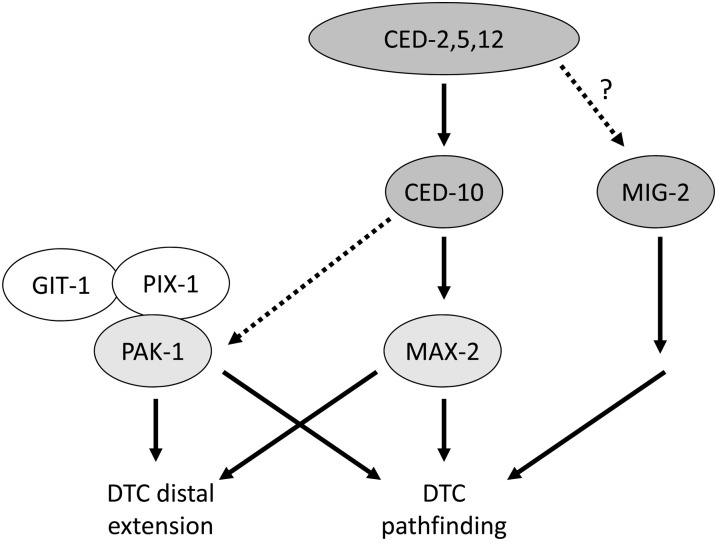
A model for Pak and Rac function in DTC migration. Loss of *max-2/Pak* and *ced-10/Rac* are strongly synthetic with loss of *pak-1/Pak*, *git-1*, and *pix-1*, which argues that these two groups of proteins function in parallel and largely redundant pathways to control DTC pathfinding and extension. In human cells, conventional group A Paks highly orthologous to *C. elegans* PAK-1 function in a complex with Git and Pix proteins. Components of the Dock/ELMO atypical RacGEF (CED-2, CED-5, and CED-12) have genetic interactions with MAX-2 and PAK-1 similar to those of CED-10, arguing that the Dock/Elmo complex promotes CED-10/Rac activity to control DTC migration. Because *ced-10* single mutants have substantial DTC migration defects, we hypothesize that CED-10 plays a modest role in activity of the putative PAK-1/GIT-1/PIX-1 complex, perhaps through targeting the complex to a specific subcellular compartment, as observed in human cells. MIG-2 likely functions in parallel to CED-10, PAK-1, and MAX-2 but may or may not function downstream of Dock/ELMO atypical RacGEF.

We have characterized two Pak pathways that are required for proper migration and extension of DTCs during gonadal development. Loss of both pathways causes essentially 100% defective gonad morphology, and results in both DTC pathfinding defects and short migrations (truncation). Apparently both general classes of phenotypes are governed by the same genetic pathways (Figure 8), and thus may be mechanistically distinct processes that share the same signaling modules.

Disruption of individual pathway branches causes little or no defect, indicating extensive redundancy between the two pathways. This twofold redundancy and the robust phenotypes when both pathways are lost allows for thorough dissection of the two pathway branches, and provides a fertile avenue for future research into Pak pathway functions.

We present a model of Pak pathway composition in DTC migration based on our genetic analyses (Figure 8). One pathway features the divergent Group A Pak, MAX-2, functioning like a canonical Group A Pak, perhaps surprising given that MAX-2 lacks Group A regulatory sequences other than the PBD. Our results in conjunction with a wealth of mammalian Pak studies (reviewed in Arias-Romero and Chernoff 2008; Bokoch 2003; Hofmann *et al.* 2004) suggest that MAX-2 is bound and activated by GTP-bound CED-10/Rac. Furthermore, in DTC migration CED-10 is activated by the Dock/ELMO RacGEF complex of CED-2/CED-5/CED-12, consistent with their similar relationship in cell corpse engulfment (Wu and Horvitz 1998). We have not identified the upstream signals that control RacGEF activation in this process, though a role for integrins has been reported (Lucanic and Cheng 2008). Our data also suggest that MIG-2/RhoG, which activates the Dock/ELMO atypical RacGEF complex in human and *C. elegans* cell corpse engulfment (deBakker *et al.* 2004), does not activate the CED-2/5/12 Dock/ELMO RacGEF complex in DTC migration. We place MIG-2/RhoG in parallel to the Paks and CED-10/Rac because of the apparent mutant additivity. Formally, MIG-2/RhoG may function downstream of the RacGEF, but this relationship is unclear. We also do not know through which effectors MAX-2 and PAK-1 signal in DTC pathfinding and extension.

The canonical PAK-1, in contrast, does not appear to have an absolute requirement for GTPase input for activity, though our analysis suggests some contribution of CED-10/Rac to PAK-1 branch activity. PIX-1 and GIT-1, orthologous to the human Pix exchange factor and Git G-protein coupled receptor kinase interactor, respectively, are equally required in the PAK-1 pathway branch, and thus we have identified a putative Pak-Pix-Git complex. Our finding that ectopic PAK-1 mutated in the Pix-binding motif can no longer rescue the *pak-1* mutant phenotype corroborated this model.

### Regulation of Pak-Pix-Git complex activity

Another group has reported conclusions consistent with some but not all of our data (Lucanic and Cheng 2008). They describe *pak-1* mutant defects in the morphology of DTCs that we were unable to replicate in blind assays. The human Pak-Pix-Git complex has been reported to function independently of kinase activity, and thus the role of Pak is thought to be that of a scaffold for nucleating the complex (Li *et al.* 2003). However, our KD PAK-1 construct failed to rescue the *pak-1* synthetic mutant phenotype compared to successful rescue by a PAK-1(+) control. Although we cannot confirm that transgenic protein is expressed, the wild-type PAK-1 did rescue the double loss of function, and the Pix- and kinase-dead have been validated in human proteins. Thus, our results argue that a functional kinase domain is required in part for full Pak-Pix-Git complex activity. Alternatively, perhaps there is more than one PAK-1-mediated activity present in DTCs. For example, a major PAK-1 function in DTCs may be that of the scaffold in the Pak-Pix-Git complex, but there is also conventional Pak kinase activity. Contradicting this model is the observation that the requirement of PIX-1 and GIT-1 are equally as strong as the requirement for PAK-1 in DTC migration (Figures 1C and 3D).

Lucanic and Cheng (2008) also weakly rescued *pak-1* gonad morphology defects, which we cannot reproduce, with a GTPase-interaction dead PAK-1 protein, suggesting that PAK-1 activities are Rac- and CDC-42-independent (Lucanic and Cheng 2008). However, our genetic data argue that CED-10/Rac contributes significantly to the PAK-1 signaling branch, raising the possibility that the Pak-Pix-Git complex is not entirely Rac-independent.

If our model were true, then the canonical PAK-1 activity would be predicted to be GTPase-dependent. Our observations suggest that CED-10 plays a moderate role in PAK-1 branch activity in addition to its strong requirement for MAX-2 branch activity. First, even hypomorphic loss of CED-10 (or the CED-2/5/12 components of the atypical RacGEF) function confers a substantial DTC migration defect, while strong loss of single Pak proteins caused little (*e.g.*, MAX-2) or no (*e.g.*, PAK-1) defect. If CED-10 signaled solely through MAX-2, then the *ced-10* loss-of-function (lf) phenotype should be comparable to that of *max-2* mutants. Second, *ced-10(*lf*)* confers a substantial truncation defect, whereas we typically see substantial truncation with loss of both pathways (see Table 2). Third, ectopically expressed activated CED-10 cannot be suppressed by loss of either *pak-1* or *max-2*, thus arguing that ectopic activated CED-10/Rac inappropriately engages both pathways. The parsimonious explanation of these observations is that CED-10 contributes to PAK-1 branch activity, and thus loss of CED-10 strongly disrupts the MAX-2 branch in addition to weakly disrupting the PAK-1 branch. However, we cannot determine whether that contribution is through targeting the Pak-Pix-Git complex to a specific subcellular compartment to facilitate its activity (Brown *et al.* 2002), or whether CED-10 additionally activates canonical PAK-1 kinase-dependent activities.

An alternative possibility to our model is that CED-10 has roles in DTC migration independent of Pak proteins, perhaps by using additional effector proteins. CED-10 has previously been shown to use diverse effectors in axon pathfinding and hypodermal enclosure (Lucanic *et al.* 2006; Lundquist *et al.* 2001; Norris *et al.* 2009; Quinn *et al.* 2008; Shakir *et al.* 2006; Shakir *et al.* 2008; Soto *et al.* 2002; Struckhoff and Lundquist 2003), and Rac is known to use multiple effectors in human cells (Karnoub *et al.* 2004).

Our observations suggest that although CDC-42 has no nonredundant contribution to DTC migration, ectopic-activated CDC-42 is capable of engaging both PAK-1 and MAX-2. While this isn’t necessarily expected of the divergent MAX-2, we predict that CDC-42 can engage the well-conserved PAK-1 as an effector in some biological processes. The PAK-1 PBD is well conserved with human Group A Pak proteins, and the CDC-42 core effector-binding region is 100% identical to that of human Cdc42 (YVPTVFDNY).

Other studies have found a modest role for RAC-2 in DTC migration (Lucanic and Cheng 2008; Lundquist *et al.* 2001), but we saw minimal or no synthetic interactions. RAC-2 is thought to have minor redundancy with CED-10 in various processes (Lundquist *et al.* 2001), but it is unclear whether RAC-2 uses the same effectors as CED-10. We note that while the core effector-binding region of CED-10 is 100% conserved with human Rac1, Rac2 and Rac3 (YIPTVFDNY), RAC-2 has nonconservative variants in two residues (YILTVFDTY), and mutation of many of these residues in human Rac1 disrupt binding of Pak and other effectors (Westwick *et al.* 1997). Thus, it is likely that RAC-2 is a divergent Rac protein that uses a somewhat-different spectrum of effectors than CED-10 and human Racs.

Finally, we find no evidence for the MIG-2/RhoG homolog functioning in either the MAX-2 or PAK-1 branch. MIG-2 clearly plays a role in regulating DTC migration, but we propose that this role is through additional pathways. The nematode cactin homolog, CACN-1, was shown genetically to interact in the MIG-2 pathway in DTC migration and functions in parallel to CED-10, PAK-1 and MAX-2, so we echo the model that MIG-2/RhoG functions in parallel to PAK-1 and MAX-2 signaling (Tannoury *et al.* 2010). By yeast two-hybrid assay MIG-2 has been found to interact with MAX-2 (Fujiki *et al.* 2010). This result in unexpected but perhaps reflects the atypical MAX-2 PBD. Beyond this yeast two-hybrid result, the defined effectors for RhoG proteins are the ELMO atypical GEF family, phosphatidylinositol 3 kinase, phospholipase D, and kinectin (deBakker *et al.* 2004; Katoh *et al.* 2000; Vignal *et al.* 2001; Wennerberg *et al.* 2002; Yamaki *et al.* 2007). The core effector-binding sequence in the Switch 2 region of Rac (YIPTVFDNY) and Cdc42 (YVPTVFDNY) are absolutely conserved between *C. elegans* and mammals, although as noted nematode RAC-2 has divergent changes. MIG-2 (YVPTVFDNY) and RhoG (YIPTVFDNY) have one conservative residue difference (V to I), making the worm MIG-2 effector binding sequence identical to Rac while the RhoG effector binding sequence is identical to Cdc42. Therefore, at the sequence level it is plausible that MIG-2 binds MAX-2, though inconsistent with our DTC migration genetics.

Two receptor systems have been shown to regulate DTC migration. Loss of integrin function disrupts DTC migration (Baum and Garriga 1997; Cram *et al.* 2003; Lee *et al.* 2001; Meighan and Schwarzbauer 2007), but loss of integrin has no additive effects when combined with loss of Pak function (Lucanic and Cheng 2008). The netrin signaling system also regulates DTC migration (Hedgecock *et al.* 1987, 1990), and loss of Paks are additive with loss of Netrins (Lucanic and Cheng 2008). These results argue that Paks function downstream of integrins, but not Netrins. No putative downstream partners of MAX-2 or PAK-1 have been identified. An RNAi screen has identified many other factors regulating DTC migration (Cram *et al.* 2006), and the roles of many are not understood.

Rac1-dependent activation of Pak1 scaffolding for PDK-Akt activity was described (Higuchi *et al.* 2008), but this study does not state whether Pix and Git are part of this scaffold. We tested RNAi-directed at the *C. elegans* orthologs of PDK and Akt, but observed no synergy with *max-2* (data not shown).

### Paks are redundant in other nematode biological processes

We found that MAX-2 and PAK-1 are also redundant for additional biological functions. These redundant Paks are required for at least one, and probably multiple diverse roles in germline development and function. Clearly sperm development is disrupted in the double loss of function, but we argue that other functions are disrupted as well to account for the entire sterile phenotype in the double loss of function.

We found a complex relationship among the *C. elegans* Pak proteins in embryonic morphogenesis. Simplistically, we hypothesize that *max-2* antagonizes the effect of the Group B Pak, *pak-2*, but embryonic morphogenesis is a very complex process involving diverse movements of differing cell types (Zhang *et al.* 2011), and in principle these proteins could even function in separate cells. The interaction and regulation of these Pak proteins in embryogenesis will be the interesting subject of future studies. However, we were surprised that one of the most complicated series of morphogenetic events in the nematode, the development of the male tail, was not perturbed by loss of *max-2* and *pak-1*. Specifically, we found that *pak-1(ok448)*/*0*; *max-2(RNAi)* and *pak-1(tm403)*/*0*; *max-2(RNAi)* males had wild-type spacing and morphology of sensory rays and other male tail structures (data not shown).

We did, however, observe moderate defects in vulval morphogenesis in *max-2*; *pak-1* double mutant animals. The vulva frequently was protruding in double-mutant adults, and in many L4 animals the “Christmas tree” invaginating vulva is asymmetrical. This result is not surprising, because CED-10 has been previously implicated in vulval morphogenesis (Kishore and Sundaram 2002), and CDC-42 has been implicated in vulval precursor cell polarity and induction (Welchman *et al.* 2007). Either could use one or both Pak proteins to propagate their signal. In addition to the potential role in cytoskeletal regulation during vulval morphogenesis, it is interesting to consider Paks as a possible MAPKKKK or MAPKKK (MAP4 kinase or MAP3 kinase), since Paks have been described as phosphorylating Raf kinase and MEK. LET-60/Ras regulates vulval fate through Raf-MEK-ERK signaling, and we found no role for PAK-1 and MAX-2 in regulation of this pathway (data not shown). However, LET-60 also regulates post-specification vulval morphogenesis through Raf-Mek signaling, and perhaps Paks regulate Raf-MEK-ERK activity in this process.

In conclusion, our study raises two substantial mechanistic questions. First, is the Pak-Pix-Git complex kinase independent? We find that DTC-directed expression of putative kinase-dead PAK-1 fails to rescue loss of *pak-1* compared with rescuing wild-type construct. This observation argues that the PAK-1 function is in part dependent on kinase activity, in contrast to reports on the mammalian Pak-Pix-Git complex. Second, phenotypes caused by loss of CED-10 or components of its RacGEF, CED-2/5/12, confer substantially stronger migration defects than loss of either PAK-1 or MAX-2 alone, suggesting that CED-10/Rac functions in both pathway branches. One interpretation of these data are that the Pak-Pix-Git complex relies to some degree on Rac activity, perhaps by membrane targeting of the complex, and on some kinase activity. Alternatively, perhaps PAK-1 performs both canonical and non-canonical functions in DTC migration. However, this interpretation is not consistent with the requirement of PIX-1 and GIT-1 activity that is equally as strong as the PAK-1 requirement.

## References

[bib1] Allen K. M. Gleeson J. G. Bagrodia S. Partington M. W. MacMillan J. C., 1998 PAK3 mutation in nonsyndromic X-linked mental retardation. Nat. Genet. 20: 25–30 9731525 10.1038/1675

[bib2] Arias-Romero L. E. Chernoff J., 2008 A tale of two Paks. Biol. Cell 100: 97–108 18199048 10.1042/BC20070109

[bib3] Baum P. D. Garriga G., 1997 Neuronal migrations and axon fasciculation are disrupted in ina-1 integrin mutants. Neuron 19: 51–62 9247263 10.1016/s0896-6273(00)80347-5

[bib4] Bienvenu T. des Portes V. McDonell N. Carrie A. Zemni R., 2000 Missense mutation in PAK3, R67C, causes X-linked nonspecific mental retardation. Am. J. Med. Genet. 93: 294–298 10946356 10.1002/1096-8628(20000814)93:4<294::aid-ajmg8>3.0.co;2-f

[bib5] Blelloch R. Anna-Arriola S. S. Gao D. Li Y. Hodgkin J., 1999 The gon-1 gene is required for gonadal morphogenesis in *Caenorhabditis elegans*. Dev. Biol. 216: 382–393 10588887 10.1006/dbio.1999.9491

[bib6] Bokoch G. M., 2003 Biology of the p21-activated kinases. Annu. Rev. Biochem. 72: 743–781 12676796 10.1146/annurev.biochem.72.121801.161742

[bib7] Brenner S., 1974 The genetics of *Caenorhabditis elegans*. Genetics 77: 71–94 4366476 10.1093/genetics/77.1.71 PMC1213120

[bib8] Brown M. C. West K. A. Turner C. E., 2002 Paxillin-dependent paxillin kinase linker and p21-activated kinase localization to focal adhesions involves a multistep activation pathway. Mol. Biol. Cell 13: 1550–1565 12006652 10.1091/mbc.02-02-0015 PMC111126

[bib9] Chen W. Chen S. Yap S. F. Lim L., 1996 The *Caenorhabditis elegans* p21-activated kinase (CePAK) colocalizes with CeRac1 and CDC42Ce at hypodermal cell boundaries during embryo elongation. J. Biol. Chem. 271: 26362–26368 8824291 10.1074/jbc.271.42.26362

[bib10] Chisholm A. D. Hardin J., 2005 Epidermal morphogenesis (December 02, 2005), *WormBook*, ed. The *C. elegans* Research Community, WormBook, doi/10.1895/wormbook.1.35.1, http://www.wormbook.org 10.1895/wormbook.1.35.1 PMC4781537 18050408

[bib11] Cram E. J. Clark S. G. Schwarzbauer J. E., 2003 Talin loss-of-function uncovers roles in cell contractility and migration in *C. elegans*. J. Cell Sci. 116: 3871–3878 12915588 10.1242/jcs.00705

[bib12] Cram E. J. Shang H. Schwarzbauer J. E., 2006 A systematic RNA interference screen reveals a cell migration gene network in *C. elegans*. J. Cell Sci. 119: 4811–4818 17090602 10.1242/jcs.03274

[bib13] Daniels R. H. Zenke F. T. Bokoch G. M., 1999 alphaPix stimulates p21-activated kinase activity through exchange factor-dependent and -independent mechanisms. J. Biol. Chem. 274: 6047–6050 10037684 10.1074/jbc.274.10.6047

[bib14] deBakker C. D. Haney L. B. Kinchen J. M. Grimsley C. Lu M., 2004 Phagocytosis of apoptotic cells is regulated by a UNC-73/TRIO-MIG-2/RhoG signaling module and armadillo repeats of CED-12/ELMO. Curr. Biol. 14: 2208–2216 15620647 10.1016/j.cub.2004.12.029

[bib15] Demarco R. S. Lundquist E. A., 2010 RACK-1 acts with Rac GTPase signaling and UNC-115/abLIM in *Caenorhabditis elegans* axon pathfinding and cell migration. PLoS Genet. 6: e1001215 21124943 10.1371/journal.pgen.1001215 PMC2987834

[bib16] Dyer J. O. Demarco R. S. Lundquist E. A., 2010 Distinct roles of Rac GTPases and the UNC-73/Trio and PIX-1 Rac GTP exchange factors in neuroblast protrusion and migration in *C. elegans*. Small GTPases 1: 44–61 21686119 10.4161/sgtp.1.1.12991 PMC3109480

[bib17] Fujiki K. Mizuno T. Hisamoto N. Matsumoto K., 2010 The *Caenorhabditis elegans* Ste20-related kinase and Rac-type small GTPase regulate the c-Jun N-terminal kinase signaling pathway mediating the stress response. Mol. Cell. Biol. 30: 995–1003 20008556 10.1128/MCB.01131-09 PMC2815562

[bib18] Gally C. Wissler F. Zahreddine H. Quintin S. Landmann F., 2009 Myosin II regulation during *C. elegans* embryonic elongation: LET-502/ROCK, MRCK-1 and PAK-1, three kinases with different roles. Development 136: 3109–3119 19675126 10.1242/dev.039412

[bib19] Gedeon A. K. Nelson J. Gecz J. Mulley J. C., 2003 X-linked mild non-syndromic mental retardation with neuropsychiatric problems and the missense mutation A365E in PAK3. Am. J. Med. Genet. A. 120A: 509–517 12884430 10.1002/ajmg.a.20131

[bib20] Greenstein D., 2005 Control of oocyte meiotic maturation and fertilization (December 28, 2005), *WormBook*, ed. The *C. elegans* Research Community, WormBook, doi/10.1895/wormbook.1.53.1, http://www.wormbook.org 10.1895/wormbook.1.53.1 PMC4781623 18050412

[bib21] Gumienny T. L. Brugnera E. Tosello-Trampont A. C. Kinchen J. M. Haney L. B., 2001 CED-12/ELMO, a novel member of the CrkII/Dock180/Rac pathway, is required for phagocytosis and cell migration. Cell 107: 27–41 11595183 10.1016/s0092-8674(01)00520-7

[bib22] Harden N. Ricos M. Ong Y. M. Chia W. Lim L., 1999 Participation of small GTPases in dorsal closure of the Drosophila embryo: distinct roles for Rho subfamily proteins in epithelial morphogenesis. J. Cell Sci. 112(Pt 3): 273–284 9885281 10.1242/jcs.112.3.273

[bib23] Hedgecock E. M. Culotti J. G. Hall D. H. Stern B. D., 1987 Genetics of cell and axon migrations in *Caenorhabditis elegans*. Development 100: 365–382 3308403 10.1242/dev.100.3.365

[bib24] Hedgecock E. M. Culotti J. G. Hall D. H., 1990 The unc-5, unc-6, and unc-40 genes guide circumferential migrations of pioneer axons and mesodermal cells on the epidermis in *C. elegans*. Neuron 4: 61–85 2310575 10.1016/0896-6273(90)90444-k

[bib25] Higuchi M. Onishi K. Kikuchi C. Gotoh Y., 2008 Scaffolding function of PAK in the PDK1-Akt pathway. Nat. Cell Biol. 10: 1356–1364 18931661 10.1038/ncb1795

[bib26] Hofmann C. Shepelev M. Chernoff J., 2004 The genetics of Pak. J. Cell Sci. 117: 4343–4354 15331659 10.1242/jcs.01392

[bib27] Horvitz H. R. Brenner S. Hodgkin J. Herman R. K., 1979 A uniform genetic nomenclature for the nematode *Caenorhabditis elegans*. Mol. Gen. Genet. 175: 129–133 292825 10.1007/BF00425528

[bib28] Kamath R. S. Martinez-Campos M. Zipperlen P. Fraser A. G. Ahringer J., 2001 Effectiveness of specific RNA-mediated interference through ingested double-stranded RNA in Caenorhabditis elegans. Genome Biol. 2: RESEARCH0002 10.1186/gb-2000-2-1-research0002 PMC17598 11178279

[bib29] Kamath R. S. Fraser A. G. Dong Y. Poulin G. Durbin R., 2003 Systematic functional analysis of the *Caenorhabditis elegans* genome using RNAi. Nature 421: 231–237 12529635 10.1038/nature01278

[bib30] Karnoub A. E. Symons M. Campbell S. L. Der C. J., 2004 Molecular basis for Rho GTPase signaling specificity. Breast Cancer Res. Treat. 84: 61–71 14999155 10.1023/B:BREA.0000018427.84929.5c

[bib31] Katoh H. Yasui H. Yamaguchi Y. Aoki J. Fujita H., 2000 Small GTPase RhoG is a key regulator for neurite outgrowth in PC12 cells. Mol. Cell. Biol. 20: 7378–7387 10982854 10.1128/mcb.20.19.7378-7387.2000 PMC86291

[bib32] Kimble J. Crittenden S. L., 2005 Germline proliferation and its control. *WormBook*, ed. The *C. elegans* Research Community, WormBook, doi/10.1895/wormbook.1.13.1, http://www.wormbook.org 10.1895/wormbook.1.13.1 PMC4781503 18050413

[bib33] Kimble J. Hirsh D., 1979 The postembryonic cell lineages of the hermaphrodite and male gonads in *Caenorhabditis elegans*. Dev. Biol. 70: 396–417 478167 10.1016/0012-1606(79)90035-6

[bib34] Kinchen J. M. Cabello J. Klingele D. Wong K. Feichtinger R., 2005 Two pathways converge at CED-10 to mediate actin rearrangement and corpse removal in *C. elegans*. Nature 434: 93–99 15744306 10.1038/nature03263

[bib35] Kishore R. S. Sundaram M. V., 2002 ced-10 Rac and mig-2 function redundantly and act with unc-73 trio to control the orientation of vulval cell divisions and migrations in *Caenorhabditis elegans*. Dev. Biol. 241: 339–348 11784116 10.1006/dbio.2001.0513

[bib36] Kumar R. Gururaj A. E. Barnes C. J., 2006 p21-activated kinases in cancer. Nat. Rev. Cancer 6: 459–471 16723992 10.1038/nrc1892

[bib37] Kutsche K. Yntema H. Brandt A. Jantke I. Nothwang H. G., 2000 Mutations in ARHGEF6, encoding a guanine nucleotide exchange factor for Rho GTPases, in patients with X-linked mental retardation. Nat. Genet. 26: 247–250 11017088 10.1038/80002

[bib38] Lee M. Cram E. J. Shen B. Schwarzbauer J. E., 2001 Roles for beta(pat-3) integrins in development and function of *Caenorhabditis elegans* muscles and gonads. J. Biol. Chem. 276: 36404–36410 11473126 10.1074/jbc.M105795200

[bib39] Li Z. Hannigan M. Mo Z. Liu B. Lu W., 2003 Directional sensing requires G beta gamma-mediated PAK1 and PIX alpha-dependent activation of Cdc42. Cell 114: 215–227 12887923 10.1016/s0092-8674(03)00559-2

[bib40] Loo T. H. Ng Y. W. Lim L. Manser E., 2004 GIT1 activates p21-activated kinase through a mechanism independent of p21 binding. Mol. Cell. Biol. 24: 3849–3859 15082779 10.1128/MCB.24.9.3849-3859.2004 PMC387736

[bib41] Lucanic M. Cheng H. J., 2008 A RAC/CDC-42-independent GIT/PIX/PAK signaling pathway mediates cell migration in C. elegans. PLoS Genet. 4: e1000269 19023419 10.1371/journal.pgen.1000269 PMC2581894

[bib42] Lucanic M. Kiley M. Ashcroft N. L’Etoile N. Cheng H. J., 2006 The *Caenorhabditis elegans* P21-activated kinases are differentially required for UNC-6/netrin-mediated commissural motor axon guidance. Development 133: 4549–4559 17050621 10.1242/dev.02648

[bib43] Lundquist E. A. Reddien P. W. Hartwieg E. Horvitz H. R. Bargmann C. I., 2001 Three C. elegans Rac proteins and several alternative Rac regulators control axon guidance, cell migration and apoptotic cell phagocytosis. Development 128: 4475–4488 11714673 10.1242/dev.128.22.4475

[bib44] Manser E. Huang H. Y. Loo T. H. Chen X. Q. Dong J. M., 1997 Expression of constitutively active alpha-PAK reveals effects of the kinase on actin and focal complexes. Mol. Cell. Biol. 17: 1129–1143 9032240 10.1128/mcb.17.3.1129 PMC231838

[bib45] McPhie D. L. Coopersmith R. Hines-Peralta A. Chen Y. Ivins K. J., 2003 DNA synthesis and neuronal apoptosis caused by familial Alzheimer disease mutants of the amyloid precursor protein are mediated by the p21 activated kinase PAK3. J. Neurosci. 23: 6914–6927 12890786 10.1523/JNEUROSCI.23-17-06914.2003 PMC6740729

[bib46] Meighan C. M. Schwarzbauer J. E., 2007 Control of *C. elegans* hermaphrodite gonad size and shape by vab-3/Pax6-mediated regulation of integrin receptors. Genes Dev. 21: 1615–1620 17606640 10.1101/gad.1534807 PMC1899471

[bib47] Nishiwaki K., 1999 Mutations affecting symmetrical migration of distal tip cells in *Caenorhabditis elegans*. Genetics 152: 985–997 10388818 10.1093/genetics/152.3.985 PMC1460665

[bib48] Norris A. D. Dyer J. O. Lundquist E. A., 2009 The Arp2/3 complex, UNC-115/abLIM, and UNC-34/Enabled regulate axon guidance and growth cone filopodia formation in *Caenorhabditis elegans*. Neural Dev. 4: 38 19799769 10.1186/1749-8104-4-38 PMC2762468

[bib49] Parnas D. Haghighi A. P. Fetter R. D. Kim S. W. Goodman C. S., 2001 Regulation of postsynaptic structure and protein localization by the Rho-type guanine nucleotide exchange factor dPix. Neuron 32: 415–424 11709153 10.1016/s0896-6273(01)00485-8

[bib50] Patel F. B. Bernadskaya Y. Y. Chen E. Jobanputra A. Pooladi Z., 2008 The WAVE/SCAR complex promotes polarized cell movements and actin enrichment in epithelia during *C. elegans* embryogenesis. Dev. Biol. 324: 297–309 18938151 10.1016/j.ydbio.2008.09.023 PMC2629559

[bib51] Quinn C. C. Pfeil D. S. Wadsworth W. G., 2008 CED-10/Rac1 mediates axon guidance by regulating the asymmetric distribution of MIG-10/lamellipodin. Curr. Biol. 18: 808–813 18499456 10.1016/j.cub.2008.04.050 PMC2702229

[bib52] Reddien P. W. Horvitz H. R., 2000 CED-2/CrkII and CED-10/Rac control phagocytosis and cell migration in *Caenorhabditis elegans*. Nat. Cell Biol. 2: 131–136 10707082 10.1038/35004000

[bib53] Savage-Dunn C., 2005 TGF-β signaling (September 9, 2005),*WormBook*, ed. The *C. elegans* Research Community, WormBook, doi/10.1895/wormbook.1.22.1, http://www.wormbook.org

[bib54] Sells M. A. Knaus U. G. Bagrodia S. Ambrose D. M. Bokoch G. M., 1997 Human p21-activated kinase (Pak1) regulates actin organization in mammalian cells. Curr. Biol. 7: 202–210 9395435 10.1016/s0960-9822(97)70091-5

[bib55] Shakir M. A. Gill J. S. Lundquist E. A., 2006 Interactions of UNC-34 Enabled with Rac GTPases and the NIK kinase MIG-15 in *Caenorhabditis elegans* axon pathfinding and neuronal migration. Genetics 172: 893–913 16204220 10.1534/genetics.105.046359 PMC1456253

[bib56] Shakir M. A. Jiang K. Struckhoff E. C. Demarco R. S. Patel F. B., 2008 The Arp2/3 activators WAVE and WASP have distinct genetic interactions with Rac GTPases in *Caenorhabditis elegans* axon guidance. Genetics 179: 1957–1971 18689885 10.1534/genetics.108.088963 PMC2516072

[bib57] Simmer F. Tijsterman M. Parrish S. Koushika S. P. Nonet M. L., 2002 Loss of the putative RNA-directed RNA polymerase RRF-3 makes *C. elegans* hypersensitive to RNAi. Curr. Biol. 12: 1317–1319 12176360 10.1016/s0960-9822(02)01041-2

[bib58] Soto M. C. Qadota H. Kasuya K. Inoue M. Tsuboi D., 2002 The GEX-2 and GEX-3 proteins are required for tissue morphogenesis and cell migrations in *C. elegans*. Genes Dev. 16: 620–632 11877381 10.1101/gad.955702 PMC155352

[bib59] Struckhoff E. C. Lundquist E. A., 2003 The actin-binding protein UNC-115 is an effector of Rac signaling during axon pathfinding in *C. elegans*. Development 130: 693–704 12506000 10.1242/dev.00300

[bib60] Tannoury H. Rodriguez V. Kovacevic I. Ibourk M. Lee M., 2010 CACN-1/Cactin interacts genetically with MIG-2 GTPase signaling to control distal tip cell migration in *C. elegans*. Dev. Biol. 341: 176–185 20188721 10.1016/j.ydbio.2010.02.025 PMC2854247

[bib61] Timmons L. Court D. L. Fire A., 2001 Ingestion of bacterially expressed dsRNAs can produce specific and potent genetic interference in *Caenorhabditis elegans*. Gene 263: 103–112 11223248 10.1016/s0378-1119(00)00579-5

[bib62] Vignal E. Blangy A. Martin M. Gauthier-Rouviere C. Fort P., 2001 Kinectin is a key effector of RhoG microtubule-dependent cellular activity. Mol. Cell. Biol. 21: 8022–8034 11689693 10.1128/MCB.21.23.8022-8034.2001 PMC99969

[bib63] Vlachos S. Harden N., 2011 Genetic Evidence for antagonism between Pak protein kinase and Rho1 small GTPase signaling in regulation of the actin cytoskeleton during Drosophila oogenesis. Genetics 187: 501–512 21098722 10.1534/genetics.110.120998 PMC3030492

[bib64] Welchman D. P. Mathies L. D. Ahringer J., 2007 Similar requirements for CDC-42 and the PAR-3/PAR-6/PKC-3 complex in diverse cell types. Dev. Biol. 305: 347–357 17383625 10.1016/j.ydbio.2007.02.022 PMC3330270

[bib65] Wennerberg K. Der C. J., 2004 Rho-family GTPases: it’s not only Rac and Rho (and I like it). J. Cell Sci. 117: 1301–1312 15020670 10.1242/jcs.01118

[bib66] Wennerberg K. Ellerbroek S. M. Liu R. Y. Karnoub A. E. Burridge K., 2002 RhoG signals in parallel with Rac1 and Cdc42. J. Biol. Chem. 277: 47810–47817 12376551 10.1074/jbc.M203816200

[bib67] Westwick J. K. Lambert Q. T. Clark G. J. Symons M. Van Aelst L., 1997 Rac regulation of transformation, gene expression, and actin organization by multiple, PAK-independent pathways. Mol. Cell. Biol. 17: 1324–1335 9032259 10.1128/mcb.17.3.1324 PMC231857

[bib68] Wu Y. C. Horvitz H. R., 1998 C. elegans phagocytosis and cell-migration protein CED-5 is similar to human DOCK180. Nature 392: 501–504 9548255 10.1038/33163

[bib69] Yamaki N. Negishi M. Katoh H., 2007 RhoG regulates anoikis through a phosphatidylinositol 3-kinase-dependent mechanism. Exp. Cell Res. 313: 2821–2832 17570359 10.1016/j.yexcr.2007.05.010

[bib70] Zand T. P. Reiner D. J. Der C. J., 2011 Ras effector switching promotes divergent cell fates in *C. elegan*s vulval patterning. Dev. Cell 20: 84–96 21238927 10.1016/j.devcel.2010.12.004 PMC3028984

[bib71] Zhang H. Landmann F. Zahreddine H. Rodriguez D. Koch M., 2011 A tension-induced mechanotransduction pathway promotes epithelial morphogenesis. Nature 471: 99–103 21368832 10.1038/nature09765

[bib72] Zhao L. Ma Q. L. Calon F. Harris-White M. E. Yang F., 2006 Role of p21-activated kinase pathway defects in the cognitive deficits of Alzheimer disease. Nat. Neurosci. 9: 234–242 16415866 10.1038/nn1630

[bib73] Zipkin I. D. Kindt R.M. Kenyon C. J., 1997 Role of a new Rho family member in cell and axon migration in C. elegans. Cell 90: 883–894 9298900 10.1016/s0092-8674(00)80353-0

